# The effects of pH on cortex and condensate formation

**DOI:** 10.1083/jcb.202603108

**Published:** 2026-10-16

**Authors:** Brooke E. Waechtler, Chujun Liu, Bethany A. Todd, Oiza Dimowo, Emma P. Morin, Jasmine Barraza, Mashal Nawabi, Constance Campbell, Douglas N. Robinson

**Affiliations:** 1 Department of Cell Biology, Johns Hopkins Medicine, Baltimore, MD, USA; 2 Department of Chemical and Biomolecular Engineering, Johns Hopkins Medicine, Baltimore, MD, USA; 3 Department of Physiology, Pharmacology and Therapeutics, Johns Hopkins Medicine, Baltimore, MD, USA; 4 Department of Medicine, Johns Hopkins Medicine, Baltimore, MD, USA; 5 Department of Oncology, Johns Hopkins Medicine, Baltimore, MD, USA

## Abstract

The actin cortex enables cells to undergo shape changes in response to mechanical and chemical cues. Although F-actin and actin-associated proteins are present throughout cells, cortical structures assemble beneath the membrane, while actin-associated proteins form condensate-like structures in the cytoplasm. The mechanism regulating the separation between cortical and cytoplasmic phases remains unclear. Since many actin-associated proteins display pH-dependent interactions, intracellular pH gradients may contribute to this phase separation. Here, we investigate how pH regulates cortex assembly and condensate formation in *Dictyostelium discoideum*. We find that altering extracellular pH affects actin-associated protein’s cortical localization and impacts their association with the actin network. Reconstitution assays indicate that cortexillin I–mediated actin crosslinking and bundling is enhanced at lower pH but can be affected by discoidin I, a weak, pH-dependent actin crosslinker. Finally, we found that the cell’s cortical region is slightly more acidic than the cytoplasm, supporting a model in which intracellular pH helps regulate the balance between condensate assembly and cortex formation.

## Teaser

Waechtler et al. discover that a pH differential between the actin cortical and cytoplasmic phases of the cell serves as one mechanism that controls actin cytoskeletal reorganization. Specifically, a pH gradient between these two phases may mediate the transition between cortex construction beneath the membrane and cytoskeletal protein condensate formation throughout the cytoplasm.

## Introduction

The cell’s ability to change shape in response to external stimuli is a fundamental process implicated in many cellular events such as cytokinesis, chemotaxis, cell motility, and migration (Kothari et al., 2017; Kretschmer and Collado, 1980; Mayor and Etienne-Manneville, 2016; Oliferenko et al., 2009; Srivastava et al., 2016). The cell employs a complex network of cytoskeletal proteins that are primed to sense and respond to these chemical and mechanical stimuli (Fletcher and Mullins, 2010; Kee et al., 2012; Schiffhauer and Robinson, 2017; Svitkina, 2018; West-Foyle and Robinson, 2012). Together, cytoskeletal proteins assemble large-scale structures under the plasma membrane to create the cell cortex (Chugh and Paluch, 2018; Salbreux et al., 2012; Svitkina, 2020). Additionally, a subset of these proteins exists throughout the cytoplasm (Kothari et al., 2019). Through these two pools of proteins (cortical and cytoplasmic), the cell constructs a control system that regulates cellular mechanics through cortex assembly, reinforcement, and disassembly, allowing the cell to change shape.

In *Dictyostelium discoideum*, the cortex consists of actin, nonmuscle myosin II (MyoII), actin crosslinkers like cortexillin I (CortI), and scaffolding proteins such as IQGAP1 and IQGAP2 (Lee et al., 2010; Srivastava and Robinson, 2015; van Haastert et al., 2018). Actin is a globular protein that polymerizes to form filaments throughout the cell to help dictate the cell’s shape (Dominguez and Holmes, 2011). MyoII is a motor protein that binds to actin and uses ATP to generate force along actin filaments. CortI is an actin crosslinker that can bind multiple actin filaments together or link actin filaments to the membrane (Cha and Jeon, 2011; Shu et al., 2012; Stock et al., 1999). Although these proteins can bind together to create the cortex, these proteins also preassemble in the cytoplasm, forming protein complexes or condensates termed contractility kits (CKs) (Kothari et al., 2019). Cells build a spectrum of types of CKs that range from mechanoresponsive to non-mechanoresponsive with ambiguous CKs residing in between (Kothari et al., 2019; Plaza-Rodríguez et al., 2022). The mechanoresponsive CK components accumulate at sites of mechanical stress and consist of MyoII, CortI, and IQGAP2. Non-mechanoresponsive CK components, consisting of MyoII, CortI, and IQGAP1, antagonize the mechanoresponsive CKs. The ambiguous CKs include both the positive regulator, IQGAP2, and the negative regulator, IQGAP1 (Plaza-Rodríguez et al., 2022). Both sets of proteins can assemble at the cell cortex, including at the cleavage furrow during cell division (Kee et al., 2012). The preassembly of distinct cytoskeletal proteins into CKs allows for quick and succinct delivery of cytoskeletal proteins to the cortex.

Additional proteins that are not typically associated with the actin cortex also interact with CKs (Liu et al., 2026; Nguyen and Robinson, 2022). To elucidate how the CKs are assembled and regulated, a proteomics analysis was conducted on two key mechanoresponsive proteins, CortI and IQGAP2. Discoidin 1 (Dsc1) was identified as a biochemical interactor of both proteins (Kothari et al., 2019) and as a genetic interactor with CortI (Robinson and Spudich, 2000). Traditionally regarded as a secreted lectin that helps form the *Dictyostelium* extracellular slime sheath by binding to glycans such as galactose (Barondes et al., 1985; Huber and O’Day, 2015), Dsc1 also resides in the cytoplasm (Alexander et al., 1992; Fukuzawa and Ochiai, 1993). Specifically, Dsc1 interacts with contractility machinery and plays an important role in cell growth, cytokinesis, and maintenance of cortical tension (Nguyen and Robinson, 2022). Specifically, Dsc1 regulates the cortical distribution and mechanoresponsiveness of CortI and promotes the assembly of mechanoresponsive CKs by stabilizing the interactions between CortI and MyoII and between CortI and IQGAP2, likely acting as a molecular “glue” that holds the CKs together (Nguyen and Robinson, 2022).

Overall, these proteins bind to each other in the cytoplasm (along with other proteins) to form CKs. When these proteins are needed to reinforce the cortex, the CKs likely disperse when approaching the membrane, allowing these proteins to bind to actin or phospholipids for assembly of the cortex. Therefore, these proteins exist within two distinct phases: the cytoplasmic phase and the cortical phase. However, the mechanism by which the cell differentiates these cortical and cytoplasmic phases remains unknown.

Additionally, actin-associated proteins’ ability to interact with each other and/or actin is affected by pH. The network elasticity of actin alone is pH-dependent, with higher elastic measurements at lower pH values. CortI’s crosslinking ability is pH-dependent, with stronger crosslinking activity at lower pH, whereas fascin suppresses changes in elasticity of actin over varying pH values (Schmoller et al., 2012). *Dictyostelium discoideum* α-actinin, an actin crosslinker, binds to actin at pH values lower than 7.0 (Simon et al., 1988). A *Dictyostelium discoideum* actin binding protein also exhibits increased actin crosslinking at lower pH values (Condeelis et al., 1982). Additionally, a low pH increases plastin–actin crosslinking and bundling, talin–actin binding, and cofilin–actin binding (Bonet et al., 2000; Runyan et al., 2025; Srivastava et al., 2008). When subjected to lower pH values, MyoII’s dwell time on actin filaments increases (Debold et al., 2008). Furthermore, lower pH causes an increase in MyoII’s ability to contract actin networks in a purified system (Köhler et al., 2012) and promotes MyoII bipolar thick filament formation (Pollard, 1982).

The cell regulates internal pH through a H^+^ ion pump, such as the sodium–hydrogen exchanger, NHE1. In migrating cells, NHE1 enriches toward the leading edge and creates a pH gradient between the cell front and cell back (Martin et al., 2011). Phosphorylation of cortactin regulates the localization of NHE1. Specifically, cortactin phosphorylation increases localized NHE1, which, in turn, increases local pH and promotes cofilin activity leading to the formation of invadopodium (Magalhaes et al., 2011). Overall, actin and actin-associated proteins’ affinity is often pH-dependent with many actin-associated proteins having increased interactions with actin at lower pH values, suggesting that intracellular pH differentials could mediate actin cortex assembly.

Furthermore, filamentous actin is present throughout both the cortical and cytoplasmic phases. Actin-associated proteins typically interact with actin filaments in the cortical phase. Nevertheless, the concentration of actin filaments throughout the cytoplasmic phase is often within a range that should permit actin-associated protein binding and cortex assembly. However, the cortical and cytoplasmic phase of the cell still persists. Since the binding affinity of many actin and actin-associated proteins is affected by pH, we hypothesize that localized changes in pH contribute to the separation between these two cytoskeletal phases.

## Results

### CortI and MyoII localization and interactions with actin are affected by pH

**Figure 1.**
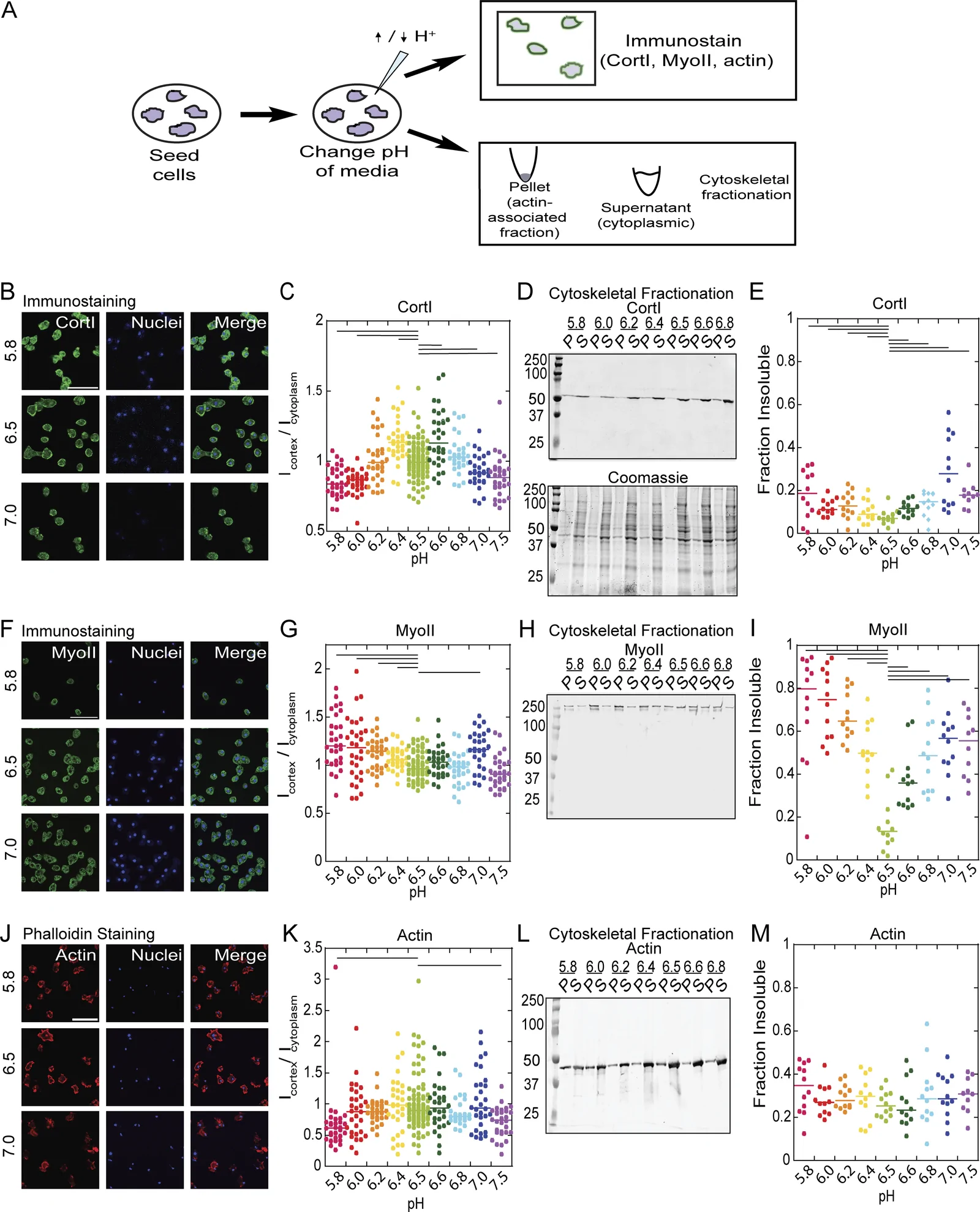
**Effects of pH on cortical localization of CortI, MyoII, and actin. (A)** Schematic of cell treatment and experiment performed (immunofluorescence, cytoskeletal fractionation assay, and fluorescence correlation spectroscopy). **(B)** Representative images of immunofluorescence imaging of CortI (green) and nuclei (blue) when incubated in media of varying pH values for 1 h. Images were collected on a confocal microscope (scale bar = 30 μm). **(C)** Quantification of CortI cortical localization by measuring the ratio of average fluorescence intensity at the cortex (I_Cortex_) to the cytoplasm (I_Cytoplasm_). Data were normalized to 6.5 by the day the slide was stained to account for any variation in day-to-day staining. **(D)** Representative western blot using mouse α-CortI– and Coomassie-stained gel for a cytoskeletal fractionation assay after cells had been incubated in varying pH media for 1 h. **(E)** Quantification of insoluble fraction of CortI by measuring integrated density of pellet and supernatant fractions. **(F)** Representative images of immunofluorescence imaging of MyoII (green) and nuclei (blue) when incubated in media of varying pH values for 1 h. Images were collected on a confocal microscope (scale bar = 30 μm). **(G)** Quantification of MyoII cortical localization by measuring the ratio of average fluorescence intensity at the cortex (I_Cortex_) to the cytoplasm (I_Cytoplasm_). Data were normalized to 6.5 by the day the slide was stained to account for any variation in day-to-day staining. **(H)** Representative western blot using mouse α-MyoII for a cytoskeletal fractionation assay after cells had been incubated in varying pH media for 1 h. **(I)** Quantification of insoluble fraction of MyoII by measuring integrated density of pellet and supernatant fractions. **(J)** Representative images of rhodamine-phalloidin–stained actin (red) and nuclei (blue) when incubated in media of varying pH values for 1 h. Images were collected on a confocal microscope (scale bar = 30 μm). **(K)** Quantification of actin cortical localization by measuring the ratio of average fluorescence intensity at the cortex (I_Cortex_) to the cytoplasm (I_Cytoplasm_). Data were normalized to 6.5 by the day the slide was stained to account for any variation in day-to-day staining. **(L)** Representative western blot using mouse α-actin for a cytoskeletal fractionation assay after cells had been incubated in varying pH media for 1 h. **(M)** Quantification of insoluble fraction of actin by measuring integrated density of pellet and supernatant fractions. All P values were obtained by performing a Kruskal–Wallis test followed by the Wilcoxon–Mann–Whitney test with bars indicating a P value <0.05. Source data are available for this figure: SourceData F1.

**Figure S1.**
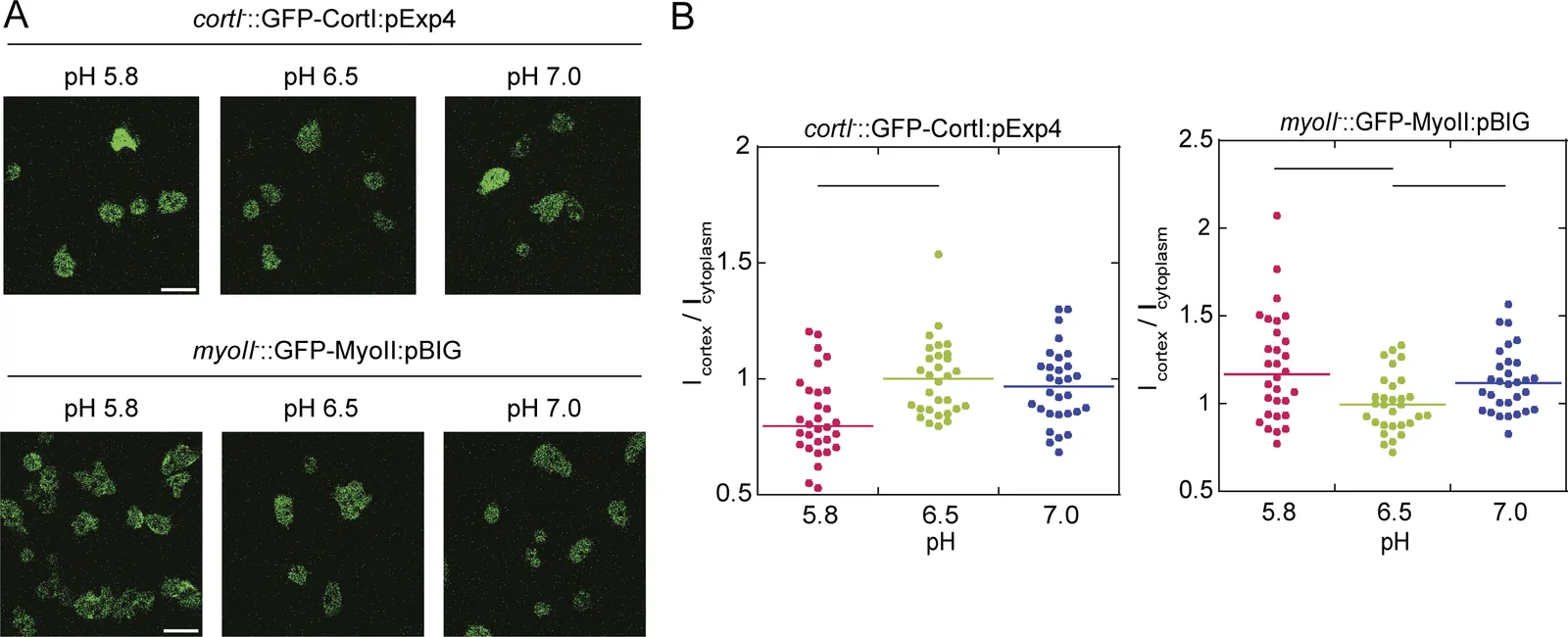
**Effects of pH on cortical localization of CortI and MyoII in live cells. (A)** Representative images *cortI*^*−*^:GFP-CortI:pExp4 and *myoII*^*−*^:GFP-MyoII:pBIG incubated in media of varying pH values for 1 h. Images were collected on a confocal microscope (scale bar = 20 μm). **(B)** Quantification of CortI and MyoII cortical localization by measuring the ratio of average fluorescence intensity at the cortex (I_Cortex_) to the cytoplasm (I_Cytoplasm_). Data were normalized to 6.5 by the day to account for any variation in day-to-day imaging. All P values were obtained by performing a Kruskal–Wallis test followed by the Wilcoxon–Mann–Whitney test with bars indicating a P value <0.05.

Since pH affects cytoskeletal proteins’ ability to interact with actin in purified systems, and the internal pH of cells is affected by external pH (Furukawa et al., 1988), we began by testing whether external pH can affect the localization of cytoskeletal proteins using immunostaining (Fig. 1 A). Specifically, wild-type (WT) cells were incubated in media of varying pH values (5.8–8.0) and were immunostained for CortI (Fig. 1 B) and MyoII (Fig. 1 F). In standard laboratory (aka axenic) media (pH 6.5) for *Dictyostelium discoideum* cell culture, CortI strongly localized to the cortex. Increasing or decreasing the pH of the media reduced CortI cortical localization, as CortI appeared more evenly distributed throughout the cell (Fig. 1 C). Conversely, MyoII had a slight increase in cortical localization when changing the pH of the media (Fig. 1 G). Additionally, filamentous actin was stained for using rhodamine-phalloidin (Fig. 1 J). A slight decrease in cortically localized actin was observed when increasing or decreasing the pH of the media (Fig. 1 K). Similar effects were observed when live rescue cell lines (*cortI*^*−*^::GFP-CortI:pExp4 and *myoII*^*−*^::GFP-MyoII:pBIG) were incubated in varying pH media (Fig. S1, A and B). Therefore, the external pH of the media influences the localization of specific cytoskeletal proteins in the cell.

**Figure S2.**
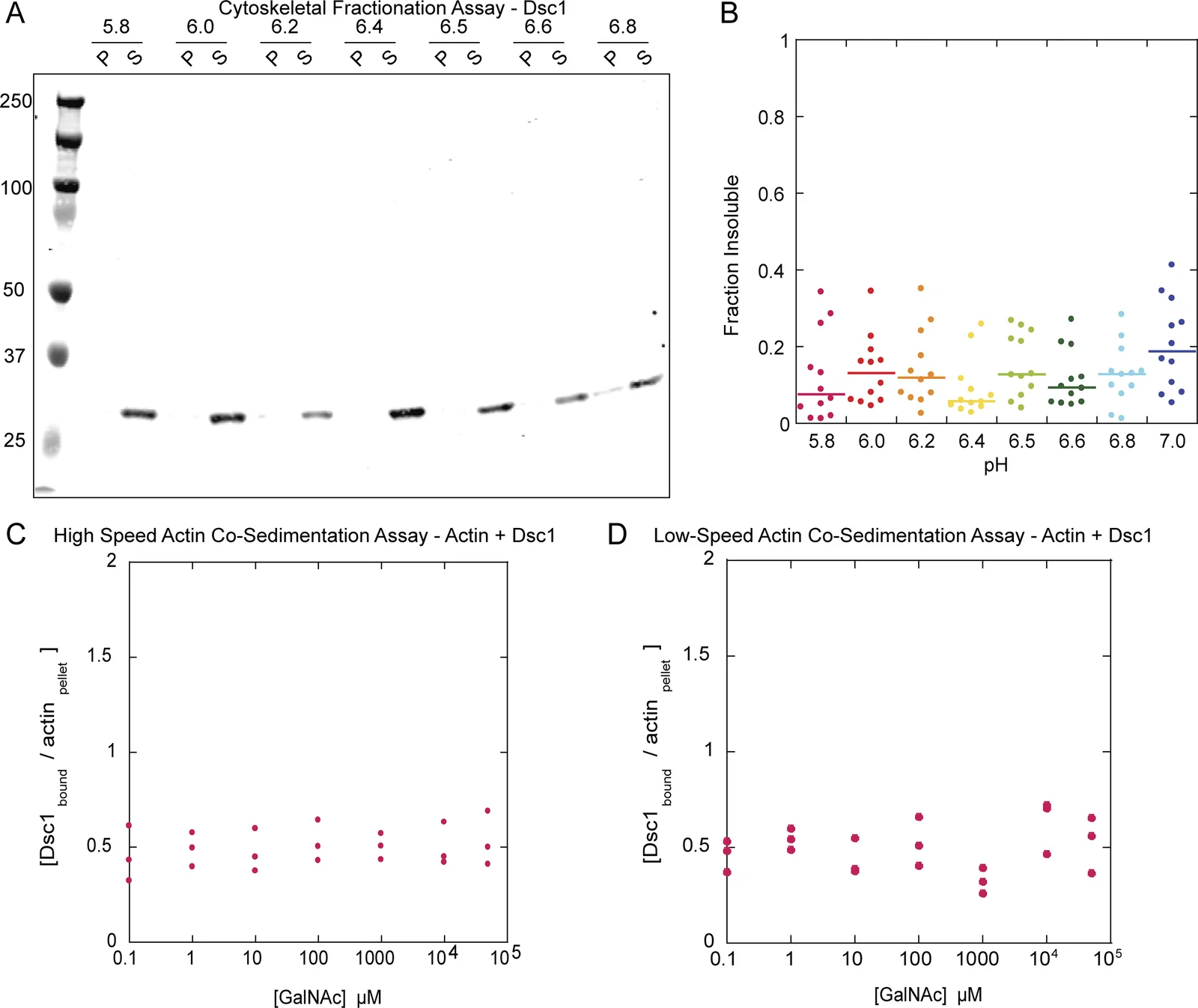
**Dsc1 cytoskeletal fractionation assay. (A)** Representative western blot using mouse α-Dsc1 of a cytoskeletal fractionation assay after cells had been incubated in varying pH values for 1 h. **(B)** Quantification of insoluble fraction of Dsc1 by measuring integrated density of pellet and supernatant fractions. **(C and D)** Quantified results for (C) high-speed and (D) low-speed actin sedimentation assays for Dsc1 in the presence of GalNAc at a pH of 6.0. No differences in binding or crosslinking and bundling between actin and Dsc1 were observed when adding GalNAc. Source data are available for this figure: SourceData FS2.

Additionally, cytoskeletal fractionation assays were used to determine how pH could affect cytoskeletal proteins’ ability to interact with actin (Fig. 1 A). Cells were incubated with media of varying pH values for 1 h, lysed, and centrifuged to sediment all crosslinked and bundled actin and associated proteins. Cells in standard pH media had the lowest fraction of insoluble CortI and MyoII; however, increasing or decreasing the pH of the media increased the insoluble fraction of these proteins (Fig. 1, D, E, H, and I). No change in solubility of actin (Fig. 1, L and M) or Dsc1 (Fig. S2, A and B) was observed. The increase in insoluble CortI and MyoII indicates that both proteins have increased interactions with actin when changing the external pH.

**Figure S3.**
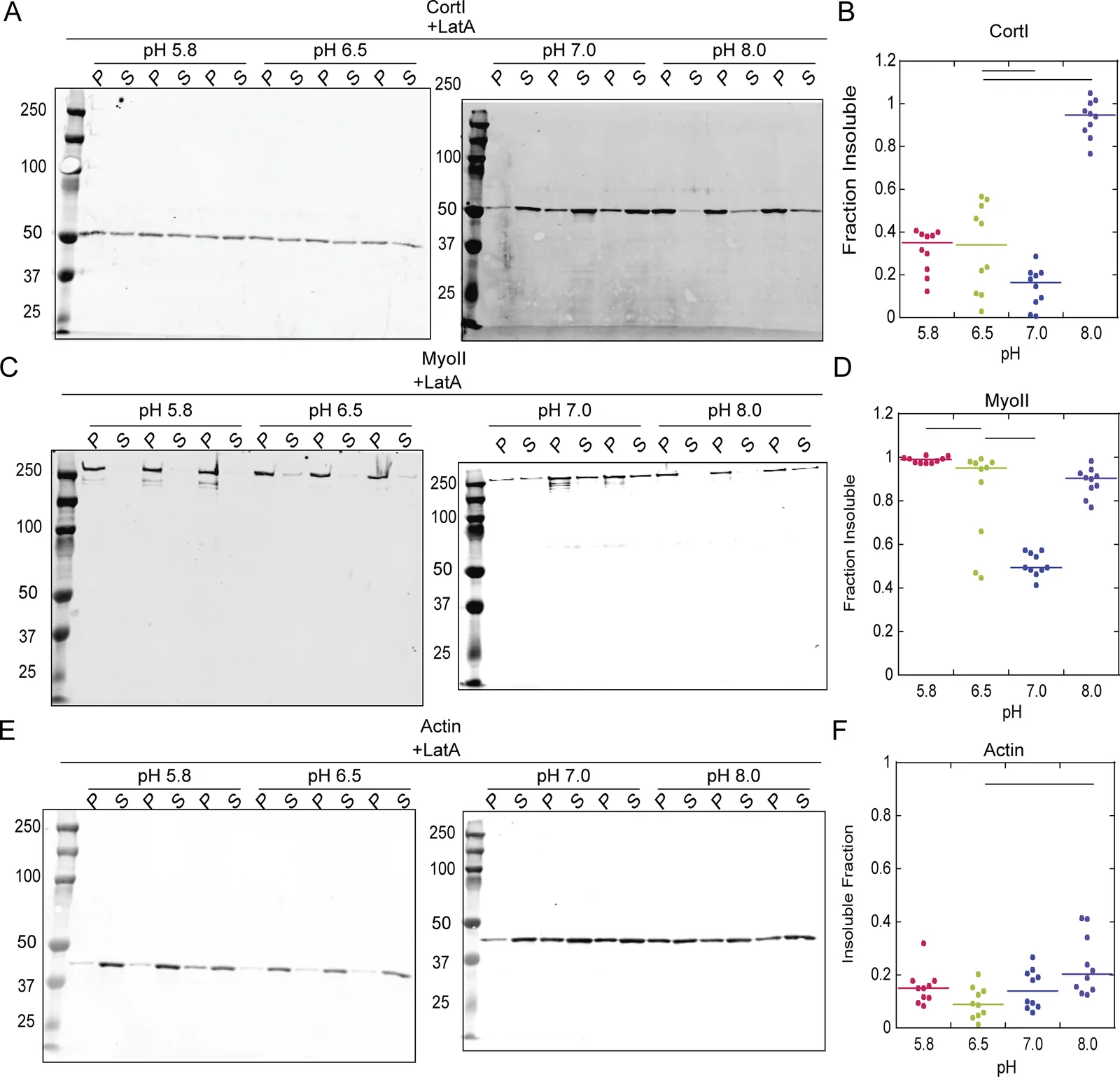
**Latrunculin A–treated cytoskeletal fractionation assays. (A)** Representative western blot using mouse α-CortI of a cytoskeletal fractionation assay after cells had been incubated in varying pH values for 1 h and treated with 5 μM latrunculin A for 30 min. **(B)** Quantification of insoluble fraction of CortI by measuring integrated density of pellet and supernatant fractions. **(C)** Representative western blot using mouse α-MyoII of a cytoskeletal fractionation assay after cells had been incubated in varying pH values for 1 h and treated with 5 μM latrunculin A for 30 min. **(D)** Quantification of insoluble fraction of MyoII by measuring integrated density of pellet and supernatant fractions. **(E)** Representative western blot using mouse α-actin of a cytoskeletal fractionation assay after cells had been incubated in varying pH values for 1 h and treated with 5 μM latrunculin A for 30 min. **(F)** Quantification of insoluble fraction of actin by measuring integrated density of pellet and supernatant fractions. All P values were obtained by performing a Kruskal–Wallis test followed by the Wilcoxon–Mann–Whitney test with bars in B, D, and F indicating a P value <0.05. Source data are available for this figure: SourceData FS3.

To verify that the observed effects were due to protein interactions with actin, cytoskeletal fractionation assays were performed after a 30-min incubation with 5 μM latrunculin A for cell incubated for 1 h in pH 5.8, 6.5, 7.0, and 8.0 media (Fig. S3). When performing these assays at a pH of 8.0, the pellet fraction looked noticeably different than pellet fractions obtained at other pH values; therefore, the effects seen at a pH of 8.0 likely reflect altered sample composition rather than changes in protein binding. In the latrunculin A–treated cells, the insoluble fraction of actin was significantly decreased, indicating that latrunculin A treatment decreased the overall actin cortical structures as expected (Fig. 1 K compared with Fig. S3 F). When latrunculin A–treated cells were incubated in pH 8.0 media, an increase in actin’s insoluble fraction was observed; however, no differences in actin insoluble fraction were observed between typical pH media (pH 6.5) and the other pH values (Fig. S3, E and F). When probing the latrunculin A–treated cells for CortI, no difference between typical media (pH 6.5) and lower pH values was observed; however, a decrease in the insoluble fraction was observed when cells were treated with pH 7.0 media (which is the opposite effect seen for the non-latrunculin A–treated cells) (Fig. S3, A and B). This indicates that the pH-dependent CortI effects observed in the non-latrunculin A–treated cells were due to increased interactions with actin. When probing for MyoII, the insoluble fraction of MyoII at a pH of 6.5 was significantly increased in the latrunculin A–treated cells compared with the non-latrunculin A–treated cells (Fig. 1 I compared with Fig. S3 D), indicating that MyoII may be forming more bipolar thick filaments after cells undergo latrunculin A treatment. Furthermore, decreasing the pH of the media (5.8) increases the insoluble fraction of MyoII, while increasing the pH of the media (7.0) decreases the insoluble fraction of MyoII (Fig. S3, C and D). No difference in the insoluble fraction of MyoII between typical media (pH 6.5) and pH 8.0 media was observed. The effects observed for MyoII insolubility do not clearly identify whether the pH-dependent MyoII effects observed in the non-latrunculin A–treated cells were due to increased interactions with actin.

Through immunostaining, we saw a loss of cortically localized CortI when cells were treated with higher and lower pH media. Simultaneously, we measured an increase in actin network formation using cytoskeletal fractionation assays. Although these results appear to contradict each other, loss of cortical localization with the increase in actin network formation could be due to a loss of the phase separation between the cortical and cytoplasmic phases. The loss in cortical–cytoplasmic phase separation could allow for CortI to bind to actin throughout both the cortex and cytoplasmic spaces. However, MyoII has an increase in both cortical localization and insoluble fraction, so a complete loss in cortical and cytoplasmic phase differentiation appears not to be occurring. Nevertheless, the pH-dependent change in the insoluble fraction of MyoII in the cytoskeletal fractionation assay is sizable, whereas the change in cortical localization is not as extreme. The difference in magnitude of these changes could suggest that MyoII also has increased interaction with actin and/or increased bipolar filament assembly in the cytoplasmic phase of the cell.

**Figure S4.**
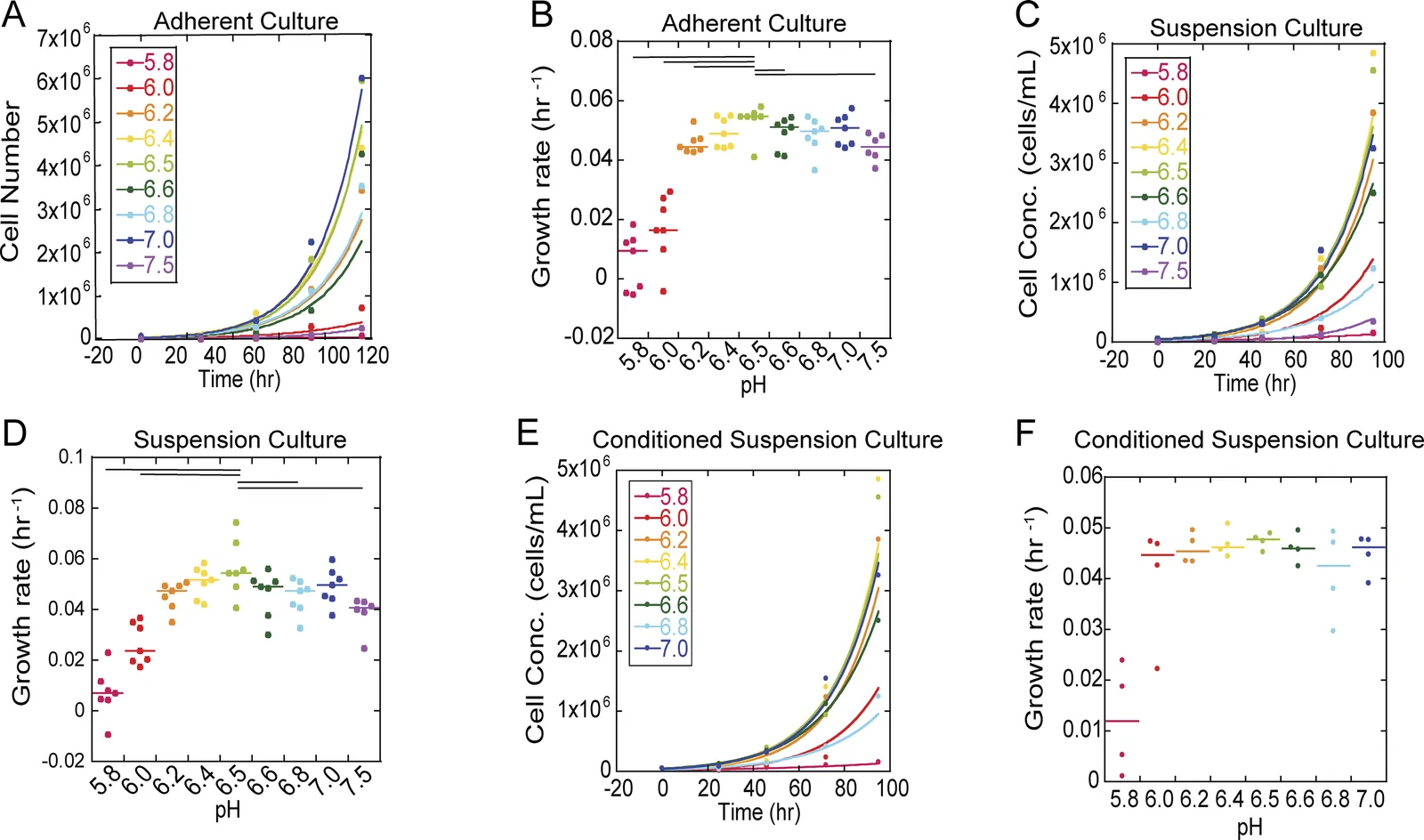
**Difference in growth rate when cells are incubated in different media. (A)** Example growth curves for KAx3 cells grown in varying pH media when cells are grown on plates. **(B)** Calculated growth rates for KAx3 cells incubated in varying media when cells are grown on plates. **(C)** Example growth curves for KAx3 cells grown in varying pH media when cells are grown in suspension. **(D)** Calculated growth rates for KAx3 cells incubated in varying media when cells are grown in suspension. **(E)** Example growth curves for KAx3 cells grown in varying pH media when cells are grown in suspension after being conditioned to the pH of the media for 1 wk. **(F)** Calculated growth rates for KAx3 cells incubated in varying media that had previously been conditioned to the pH of media for 1 wk. All P values were obtained by performing a Kruskal–Wallis test followed by the Wilcoxon–Mann–Whitney test with bars indicating a P value <0.05.

To see how the pH of the media affected the cells over time, a growth curve was created for WT cells at each pH for cells plated on a 10-cm petri dish and for cells grown in suspension culture (Fig. S4, A and C). At the lowest pH values (5.8–6.2), the growth rate was greatly reduced. At the higher pH values, the growth rate was reduced although not to the same magnitude (Fig. S4, B and D). To determine whether the cells could adapt to the pH of the growth media, cells were incubated in varying pH media for 1 wk, and growth curves were created again (Fig. S4 E). The difference in growth rates was significantly reduced or lost after being conditioned to the media, indicating that the cells can adapt to changes in pH over time (Fig. S4 F).

### Changing the external pH of the media affects cellular shape and random migration

**Figure 2.**
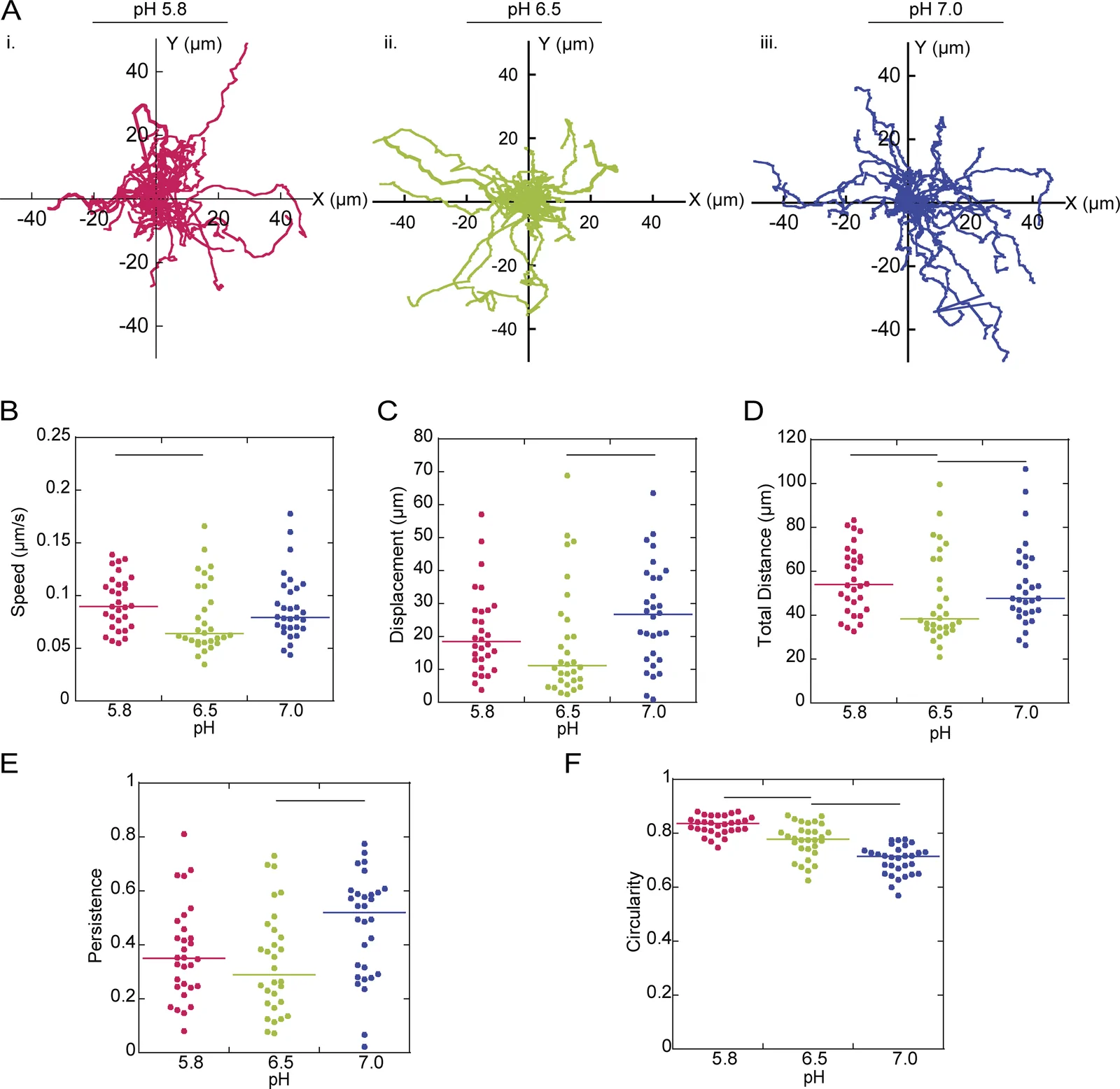
**Effects of pH on cellular shape and migration. (A)** Individual cell movement tracks for pH 5.8, 6.5, and 7.0. Each line represents a signal cell. **(B–F)** Quantification of cellular (B) speed, (C) displacement, (D) total distance, (E) persistence, and (F) circularity. All P values were obtained by performing a Kruskal–Wallis test followed by the Wilcoxon–Mann–Whitney test with bars indicating a P value <0.05.

Since differences in cytoskeletal protein localization and actin network formation were observed when changing the external pH of the media, we further assessed how external pH affects cell migration and cell shape. WT cells were incubated in varying pH media for 1 h, and videos of the cells were taken to assess random cell migration (Fig. 2 A). Decreasing the pH of the media led to an increase in cellular speed (Fig. 2 B) and total distance traveled (Fig. 2 D). Increasing the pH of the media led to an increase in cell displacement (Fig. 2 C), total distance traveled (Fig. 2 D), and cell persistence (Fig. 2 E). Overall, changing the pH of the media results in increases in all random cell migration parameters, indicating an overall increase in cell migration.

Using the same random cell migration data, cell circularity was also measured as an indicator of cell shape. Decreasing the pH of the media resulted in an increase in cell circularity; however, increasing the pH of the media resulted in a decrease in cell circularity (Fig. 2 E). Therefore, changing the external pH results in a change in cell shape as measured by cell circularity.

### CortI’s ability to crosslink and bundle actin is dependent on pH and Dsc1 presence

**Figure S5.**
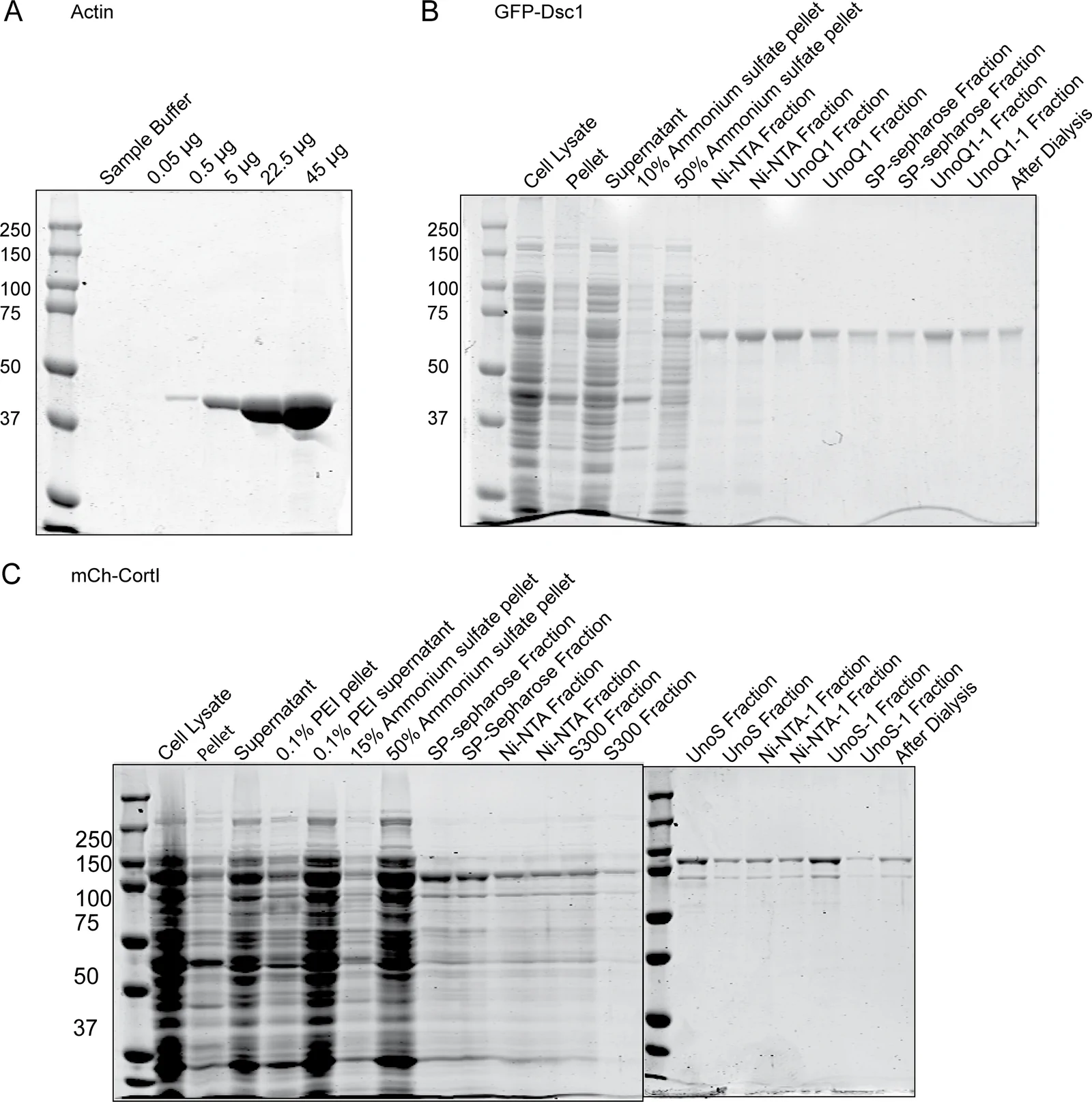
**Protein purification products. (A)** Coomassie-stained, 10% SDS-PAGE gel for purified actin. **(B and C)** Coomassie-stained, 10% SDS-PAGE gels for each step of protein purification for (B) GFP-Dsc1 and (C) mCh-CortI. Source data are available for this figure: SourceData FS5.

**Figure S6.**
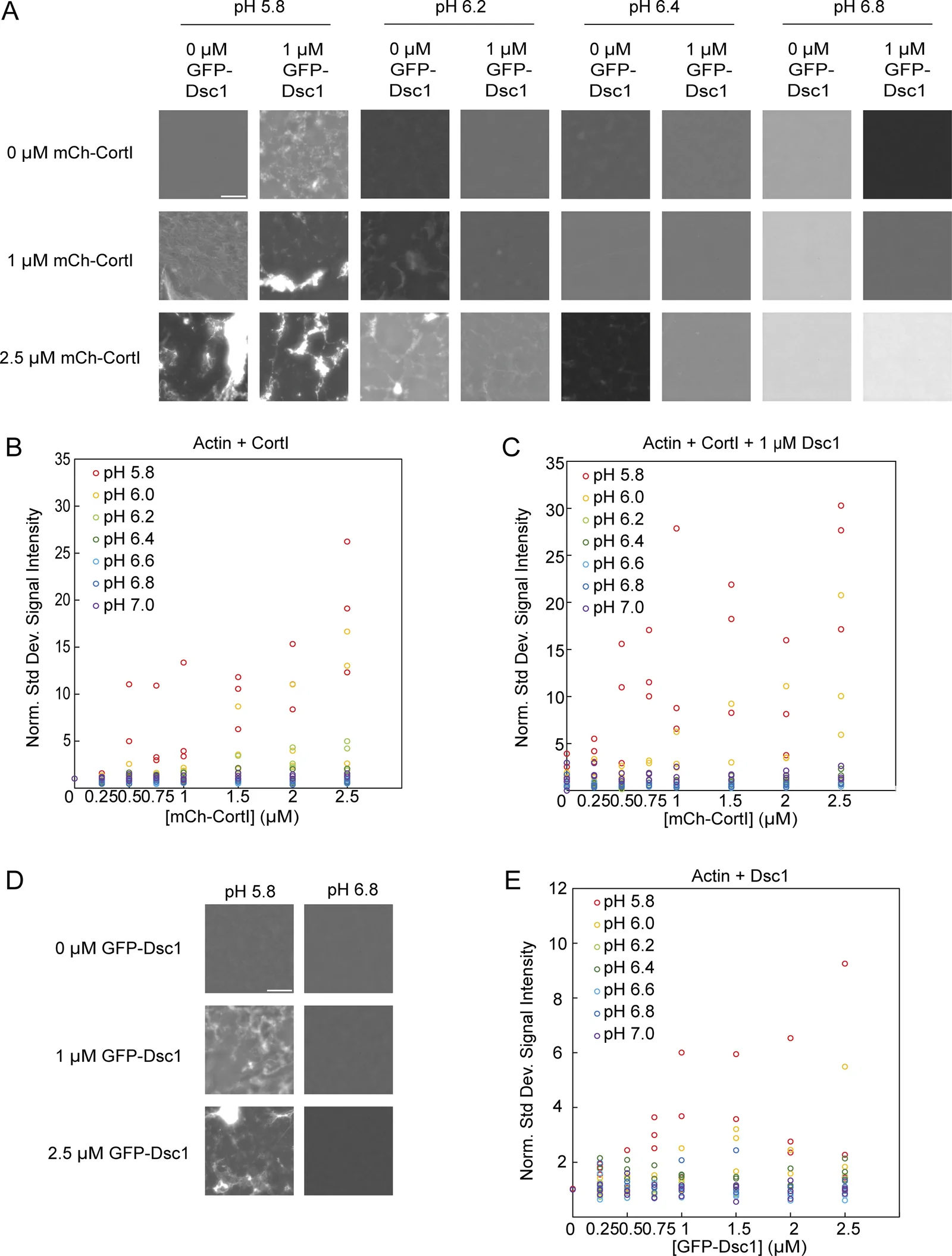
**High-throughput imaging assay to assess actin crosslinking and bundling by CortI and/or Dsc1 at varying pH values. (A)** Representative images of 647-phalloidin–stained actin over a range of pH values and concentrations of mCh-CortI with and without GFP-Dsc1 (scale bar = 100 μm). **(B)** Quantification of the standard deviation of 647-nm channel to assay actin crosslinking and bundling by mCh-CortI in varying pH values. **(C)** Quantification of the standard deviation of 647-nm channel to assay actin crosslinking and bundling by mCh-CortI in varying pH values when 1 μM GFP-Dsc1 is added. **(D)** Representative images of 647-phalloidin–stained actin over a range of pH values and concentrations of GFP-Dsc1 (scale bar = 100 μm). **(E)** Quantification of the standard deviation of 647-nm channel to assay actin crosslinking and bundling by GFP-Dsc1 in varying pH values.

CortI is an actin crosslinker that can bundle actin together or attach actin to the membrane via interaction with phosphatidylinositol 4,5-bisphosphate (Luo et al., 2014). Additionally, *dsc1*^*−*^ cells have a reduction in cortically localized CortI (Nguyen and Robinson, 2022). Therefore, we tested to see whether Dsc1 can affect CortI crosslinking and bundling at a variety of pH values in a controlled system. To do this, mCherry (mCh)-CortI and GFP-Dsc1 were purified from *Escherichia coli*, and actin was purified from chicken skeletal muscle (Fig. S5). The proteins were mixed on a 96-well plate, covered with a coverslip, sealed with vacuum grease, and imaged. Protein–actin crosslinking/bundling was assessed by measuring the standard deviation of actin stained with fluorescently labeled phalloidin (Fig. S6). In samples where actin is not crosslinked and bundled, the standard deviation is low as the fluorescent signal is evenly distributed throughout the image. When actin is crosslinked and bundled, fluorescently tagged actin localizes together to create distinct bundles, thereby causing an increase in the measured standard deviation. Through this assay, mCh-CortI crosslinked and bundled actin extremely well at a pH of 5.8 and 6.0 and still crosslinked and bundled actin at a pH of 6.2 (Fig. S6, A and B). At a pH of 6.4 or higher, minimal-to-no actin crosslinking and bundling was observed. The addition of GFP-Dsc1 to actin and mCh-CortI caused an increase in actin crosslinking and bundling at a pH of 5.8 and 6.0; however, the addition of GFP-Dsc1 caused a decrease in actin crosslinking and bundling at a pH of 6.2 (Fig. S6 C). However, the addition of GFP-Dsc1 did not affect actin crosslinking and bundling at a pH of 6.4 or higher.

At a pH of 5.8 and 6.0, actin crosslinking and bundling was observed when only GFP-Dsc1 and actin were incubated together, indicating that GFP-Dsc1 is a weak actin crosslinker itself (Fig. S6, D and E). The addition of N-acetylgalactosamine (GalNAc), the monosaccharide that Dsc1 binds most strongly (Mathieu et al., 2010), did not affect actin and GFP-Dsc1 binding or crosslinking and bundling (Fig. S2, C and D); therefore, the lectin binding site unlikely interacts with actin. Since GFP-Dsc1 can crosslink and bundle actin at low pH values, it is likely that the addition of GFP-Dsc1 to the mCh-CortI and actin reaction increases actin crosslinking and bundling through an additive effect. However, at a pH of 6.2, GFP-Dsc1 cannot bind to actin but could form CK-like structures with mCh-CortI, inhibiting mCh-CortI’s ability to crosslink and bundle actin.

**Figure S7.**
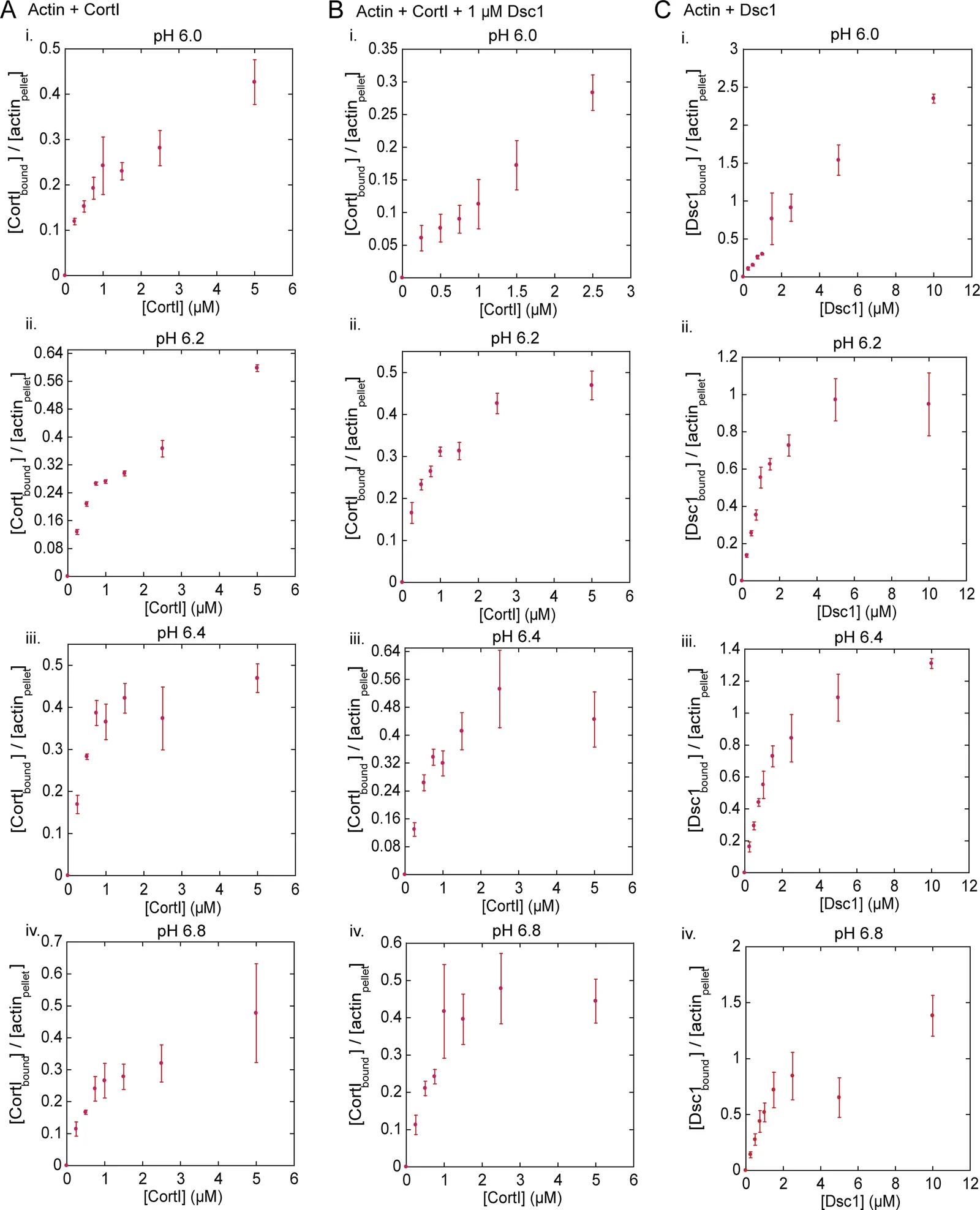
**Low-speed actin sedimentation assay curves at varying pH values. (A–C)** Low-speed actin cosedimentation assay curve between actin and crosslinkers: (A) mCh-CortI, (B) mCh-CortI with 1 μM GFP-Dsc1, and (C) GFP-Dsc1 at a pH of (i) 6.0, (ii) 6.2, (iii) 6.4, and (iv) 6.8 (average of n = 5 per binding curve; bars represent the SEM).

Once the conditions in which CortI and Dsc1 interact with actin were narrowed down, low-speed actin cosedimentation assays were performed to assess crosslinking and bundling between actin and CortI and/or Dsc1 (Heier et al., 2017). In this assay, the insoluble fraction of actin indicates the proportion of actin that has undergone crosslinking and bundling. The insoluble fraction of the mCh-CortI and/or GFP-Dsc1 is the proportion of these proteins that are bound in the crosslinked and bundled actin network. We created crosslinking/bundling curves for pH 6.0, 6.2, 6.4, and 6.8 (Fig. S7) that confirmed that CortI-mediated actin crosslinking and bundling is pH-dependent. Furthermore, CortI-mediated actin crosslinking and bundling can be Dsc1-dependent in certain pH conditions. Additionally, the low-speed actin cosedimentation assays confirmed that Dsc1 is a weak actin crosslinker.

**Figure 3.**
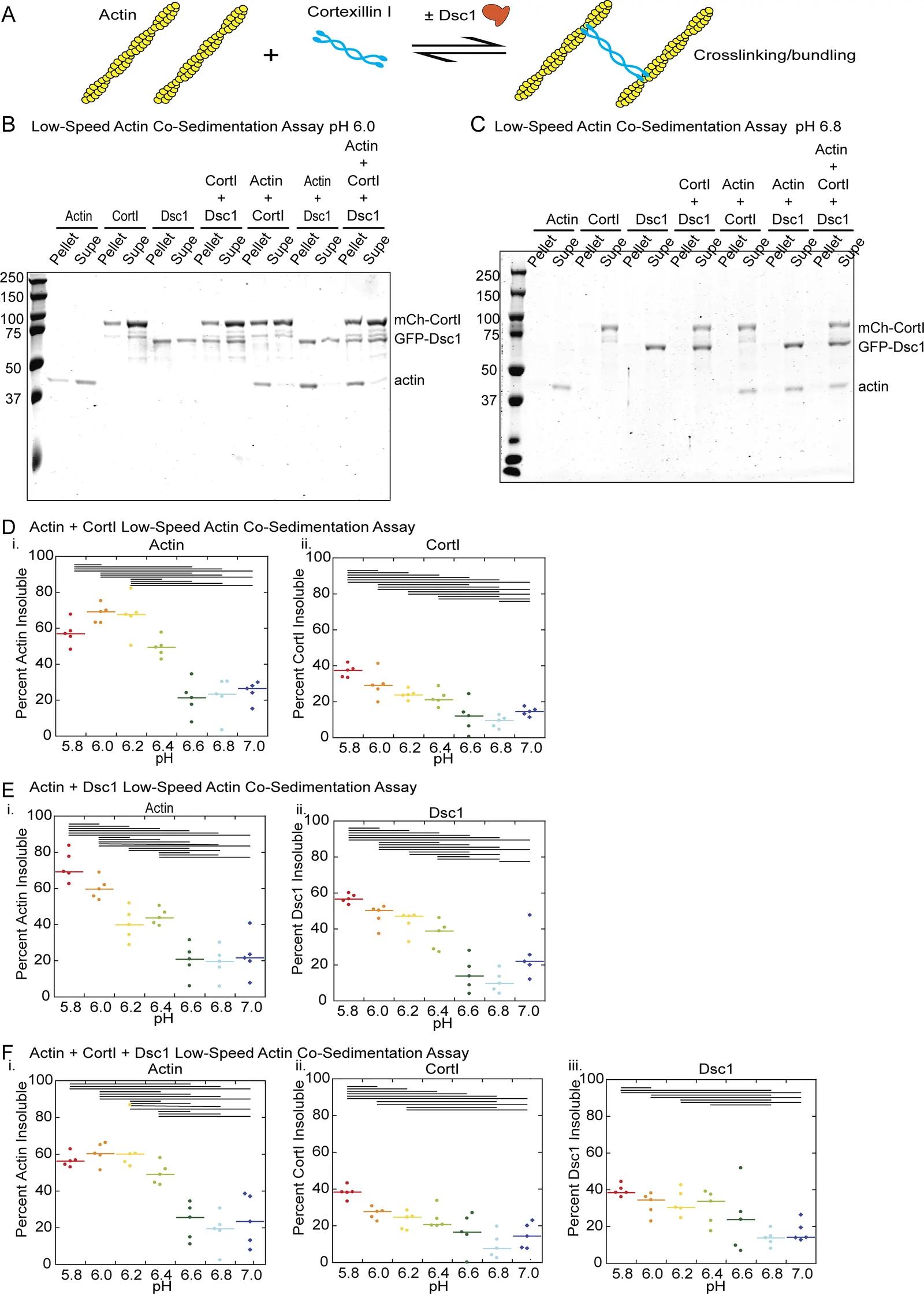
**Crosslinking and bundling of actin by mCh-CortI and GFP-Dsc1 at varying pH values. (A)** Schematic depicting crosslinker-mediated actin crosslinking and bundling. **(B and C)** Representative Coomassie-stained, 10% SDS-PAGE gels for a low-speed actin cosedimentation assay at pH (B) 6.0 and (C) 6.8. Samples contain 2 μM actin with either 1 μM mCh-CortI or 1 μM GFP-Dsc1. **(D)** i. Low-speed actin cosedimentation assay between actin and mCh-CortI indicates that actin insolubility is highest at lower pH values. ii. Low-speed actin cosedimentation assay between actin and mCh-CortI indicates mCh-CortI insolubility is higher at lower pH values. **(E)** i. Low-speed actin cosedimentation assay between actin and GFP-Dsc1 indicates that actin insolubility is highest at lower pH values. ii. Low-speed actin cosedimentation assay between actin and GFP-Dsc1 indicates that GFP-Dsc1 insolubility is highest at lower pH values. **(F)** i. Low-speed actin cosedimentation assay between actin and mCh-CortI with the addition of 1 μM GFP-Dsc1 indicates that actin insolubility is highest at lower pH values. ii. Low-speed actin cosedimentation assay between actin and mCh-CortI with 1 μM GFP-Dsc1 I indicates that mCh-CortI insolubility is highest at lower pH values. iii. Low-speed actin cosedimentation assay between actin and mCh-CortI with 1 μM GFP-Dsc1 indicates that GFP-Dsc1 insolubility is highest at lower pH values. All P values were obtained by performing a Kruskal–Wallis test followed by the Wilcoxon–Mann–Whitney test with bars indicating a P value <0.05. Source data are available for this figure: SourceData F3.

We next performed low-speed actin cosedimentation assays at all previously tested pH values with 2 μM actin, 1 μM mCh-CortI, and/or 1 μM GFP-Dsc1 (Fig. 3 A). At lower pH values, the addition of mCh-CortI or GFP-Dsc1 has a higher percent insoluble actin compared with lower pH values, indicating that these proteins can crosslink and bundle actin better at lower pH values (Fig. 3, B–Di, and Ei). Additionally, the insoluble fractions of both mCh-CortI and GFP-Dsc1 are increased at lower pH values, indicating that these proteins are crosslinking and bundling actin themselves (Fig. 3, Dii and Eii). The addition of GFP-Dsc1 appears to mildly decrease actin crosslinking and bundling at a pH of 6.0 and 6.2, as the insoluble fraction of actin decreases (Fig. 3 Fi compared with Fig. 3 Di); however, the insoluble fraction of mCh-CortI does not change (Fig. 3 Fii compared with Fig. 3 Dii). Even in the presence of mCh-CortI, GFP-Dsc1 is still pH-sensitive with a higher fraction of insoluble GFP-Dsc1 at lower pH values compared with higher pH values (Fig. 3 Fiii). Overall, actin crosslinking and bundling by mCh-CortI and GFP-Dsc1 is increased at lower pH values. Since actin crosslinkers typically localize to the cortex region of the cell, the pH of the cortex could be regulated to increase the binding between crosslinkers and actin.

### CortI’s binding affinity to actin is dependent on pH and Dsc1 presence

**Figure 4.**
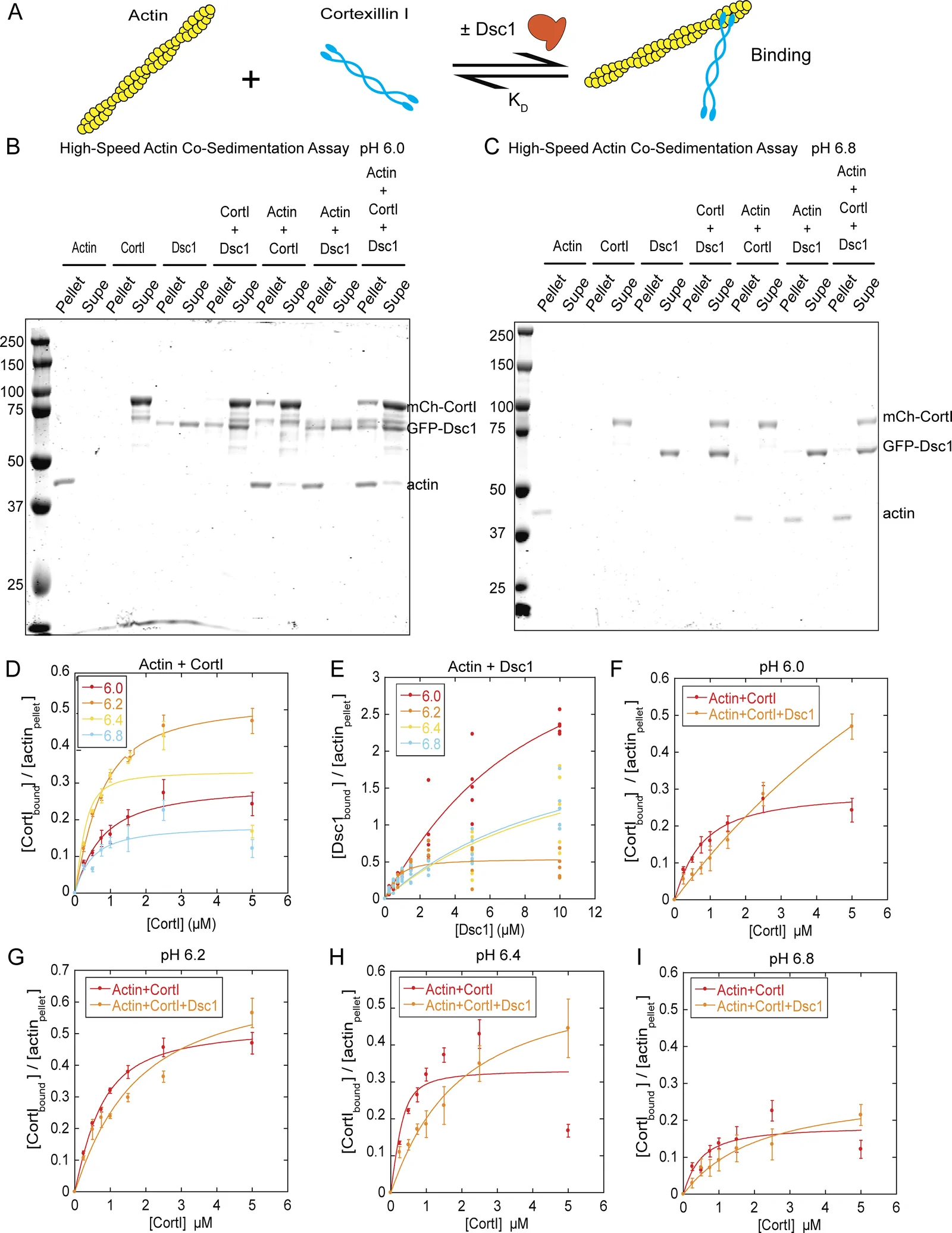
**Binding between actin and crosslinking proteins. (A)** Schematic depicting crosslinker-mediated actin crosslinking and bundling. **(B and C)** Representative Coomassie-stained, 10% SDS-PAGE gels for a high-speed actin cosedimentation assay at pH (B) 6.0 and (C) 6.8. Samples contain 2 μM actin with either 1 μM mCh-CortI or 1 μM GFP-Dsc1. **(D)** Binding curve between actin and mCh-CortI over a range of pH values (average of n = 5 per binding curve; bars represent the SEM). **(E)** Binding curves between actin and GFP-Dsc1 over a range of pH values. **(F–I)** Binding curves for actin and mCh-CortI when 1 μM GFP-Dsc1 is added over a range of pH values: (F) pH 6.0, (G) pH 6.2, (H) pH 6.4, (I) pH 6.8 (average of n = 5 per binding curve; bars represent the SEM). All actin and mCh-CortI curves from D were represented again in red to compare these curves to the curves with the addition of 1 μM GFP-Dsc1. Source data are available for this figure: SourceData F4.

**Figure S8.**
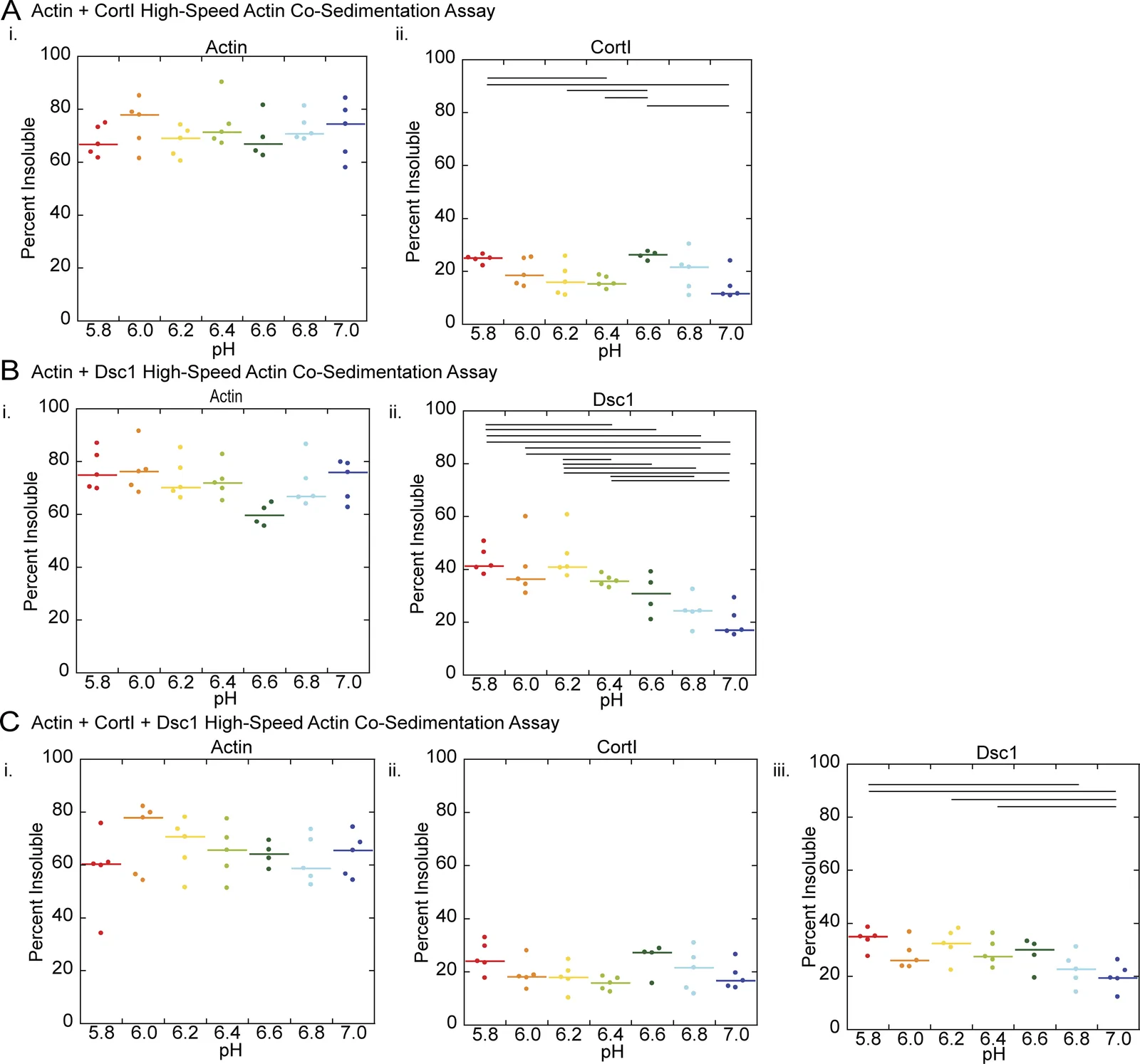
**Binding between actin and mCh-CortI or GFP-Dsc1 at varying pH values. (A)** i. High-speed actin cosedimentation assay with 2 μM actin and 1 μM mCh-CortI indicates that actin insolubility is not affected by the pH. ii. High-speed actin cosedimentation assay with 2 μM actin and 1 μM mCh-CortI indicates that mCh-CortI insolubility is higher at lower pH values. **(B)** i. High-speed actin cosedimentation assay between actin and GFP-Dsc1 indicates that actin insolubility is consistent across pH values. ii. High-speed actin cosedimentation assay with 2 μM actin and 1 μM GFP-Dsc1 indicates that Dsc1 insolubility is highest at lower pH values. **(C)** i. High-speed actin cosedimentation with 2 μM actin, 1 μM mCh-CortI, and 1 μM GFP-Dsc1 indicates that actin insolubility is constant across pH values. ii. High-speed actin cosedimentation with 2 μM actin, 1 μM mCh-CortI, and 1 μM GFP-Dsc1 indicates that CortI insolubility is not highly affected. iii. High-speed actin cosedimentation with 2 μM actin, 1 μM mCh-CortI, and 1 μM GFP-Dsc1 indicates that Dsc1 insolubility is highest at lower pH values. All P values were obtained by performing a Kruskal–Wallis test followed by the Wilcoxon–Mann–Whitney test with bars indicating a P value <0.05.

Since actin crosslinking and bundling was affected by both pH and GFP-Dsc1 addition, high-speed actin cosedimentation assays were used to assess binding between mCh-CortI and GFP-Dsc1 (Fig. 4 A). In the high-speed actin cosedimentation assay, all filamentous actin will pellet out of solution when centrifuged at 100,000 × *g*. Typically, other smaller proteins do not have a significant fraction of the protein pellet out of solution by itself. However, if a protein binds to actin, the protein will then precipitate out of solution. Using this assay, binding curves were created to determine the binding between mCh-CortI and actin at pH 6.0, 6.2, 6.4, and 6.8 (Fig. 4, B–D). The concentration of the actin-associated protein was plotted against the fraction of that protein bound over the fraction of pelleted actin, and the points were fitted with a quadratic equation to calculate the K_D_. Based on the calculated K_D_, the binding affinity between actin and mCh-CortI was the highest at a pH of 6.4 and decreased at higher and lower pH values (Table 1). Although the calculated K_D_ between actin and mCh-CortI is lowest at 6.4, the binding between actin and mCh-CortI has a pH-dependent relationship where the fraction of insoluble CortI is higher on average at lower pH values (Fig. S8 Aii).

**Table 1.** Calculated K_D_ in micromolar values for actin and mCh-CortI, actin and mCh-CortI with the addition of 1 μM GFP-Dsc1 (apparent K_D_), and actin and GFP-Dsc1 over a range of pH values

​	pH 6.0	pH 6.2	pH 6.4	pH 6.8
Actin + CortI	0.58 ± 0.17	0.44 ± 0.05	0.10 ± 0.07	0.36 ± 0.31
Actin + CortI + 1 μM Dsc1	10.8 ± 3.59	1.29 ± 0.45	1.53 ± 0.37	1.93 ± 0.54
Actin + Dsc1	5.16 ± 1.33	0.35 ± 0.21	9.25 ± 5.71	8.60 ± 4.15

To assess how GFP-Dsc1 affects binding between actin and mCh-CortI, the high-speed cosedimentation assay was repeated in the presence of 1 μM GFP-Dsc1. The addition of GFP-Dsc1 increased the apparent K_D_ between actin and mCh-CortI (Table 1), indicating a lower binding affinity between actin and mCh-CortI at all pH values when GFP-Dsc1 is present (Fig. 4, E–I). It is important to note that the apparent K_D_ reported is between actin and mCh-CortI and is calculated in a pairwise manner; however, more than two proteins are present. The insoluble fraction of mCh-CortI (the amount binding to actin) does significantly change in the presence of GFP-Dsc1. Therefore, GFP-Dsc1 is likely inhibiting mCh-CortI’s ability to bind to actin.

When performing these assays with 2 μM actin, 1 μM mCh-CortI, and 1 μM GFP-Dsc1, the insoluble fraction of mCh-CortI is on average higher at lower pH values (Fig. S8 C), once again revealing that binding between actin and mCh-CortI is pH-dependent. However, no significant change is observed in the insoluble fraction of mCh-CortI when 1 μM GFP-Dsc1 is added. The concentration of mCh-CortI in these assays is significantly higher than the calculated K_D_ between actin and mCh-CortI; therefore, mCh-CortI solubility not being affected is unsurprising.

Additionally, these high-speed actin cosedimentation assays were again performed to assay for binding between actin and GFP-Dsc1. Actin binding curves for GFP-Dsc1 were created for pH 6.0, 6.2, 6.4, and 6.8 (Fig. 4 E). When calculating the K_D_ between actin and Dsc1, the binding between these two proteins is lowest at a pH of 6.2 (Table 1). When performing these assays with 2 μM actin, 1 μM mCh-CortI, and 1 μM GFP-Dsc1, the insoluble fraction of GFP-Dsc1 is on average higher at lower pH values (Fig. S8 B), indicating that Dsc1 itself can bind to actin when the pH of the solution is lower.

### CortI and Dsc1 form CK-like structures at low pH values

**Figure 5.**
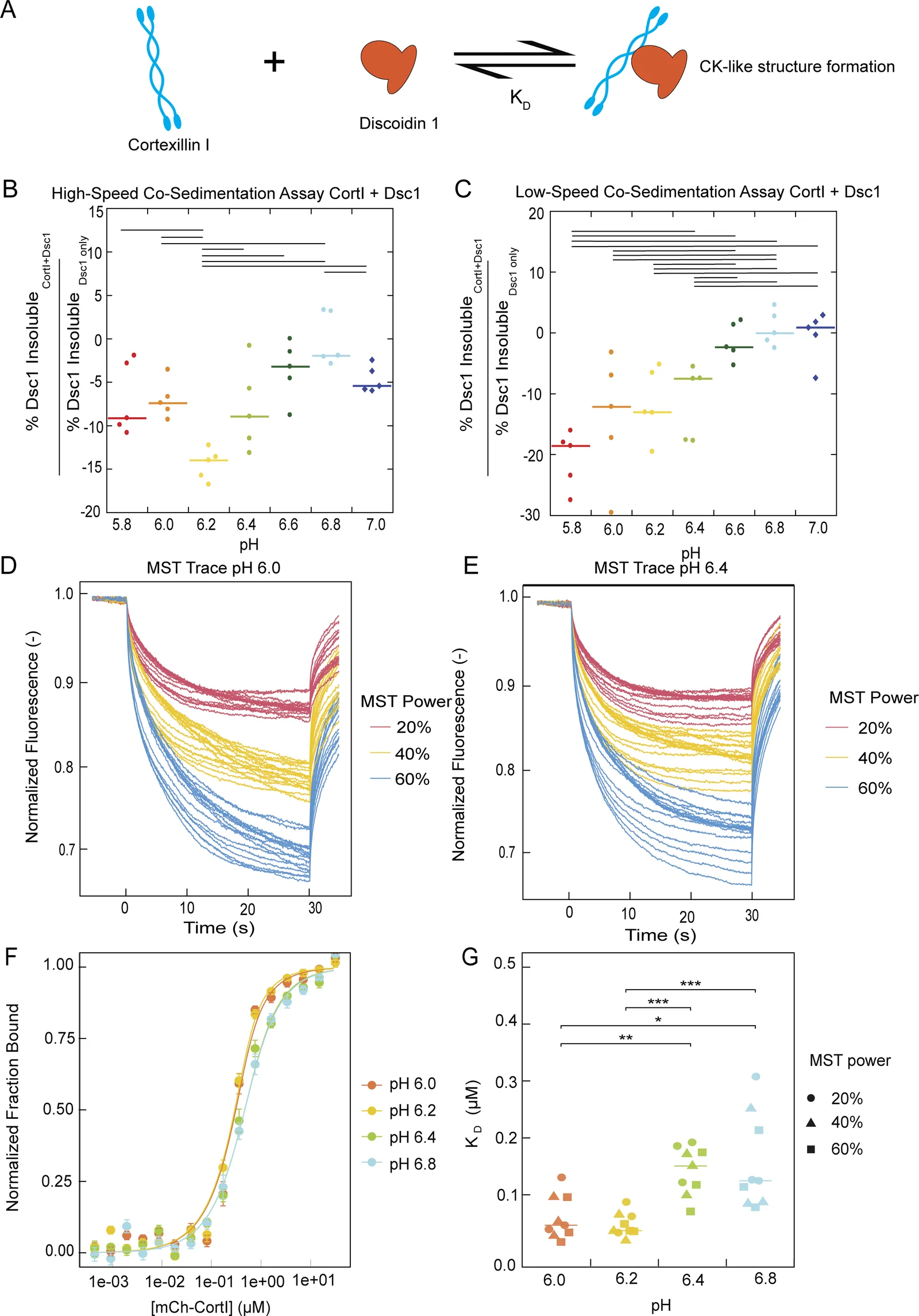
**Binding between CortI and Dsc1. (A)** Schematic depicting CortI and Dsc1 binding. **(B)** Quantified solubility of GFP-Dsc1 across pH 5.8–7.0 in the high-speed cosedimentation assay in the presence of mCh-CortI. At a pH of 6.2, adding mCh-CortI to GFP-Dsc1 increases the solubility of GFP-Dsc1, indicating binding between the two proteins. **(C)** Quantified solubility of GFP-Dsc1 across pH values 5.8–7.0 in the crosslinking and bundling assay in the presence of mCh-CortI. Solubility of GFP-Dsc1 decreases at lower pH values, indicating low-speed cosedimentation between the two. **(D and E)** MST traces measuring mCh-CortI and GFP-Dsc1 binding at 20%, 40%, and 60% MST power levels at a pH of (D) 6.0 and (E) 6.4. **(F)** Normalized fraction bound of GFP-Dsc1 to mCh-CortI at different mCh-CortI concentrations at pH 6.0, 6.2, 6.4, and 6.8. Each curve comes from an average of MST measurements at all MST power levels (average of n = 9 per binding curve; bars represent the SEM). Binding occurs at all measured pH values, with higher binding affinities at the lower pH values. **(G)** Quantified apparent K_D_ of GFP-Dsc1 to mCh-CortI at pH 6.0, 6.2, 6.4, and 6.8 from the MST measurements. Lower apparent K_D_ at pH 6.0 and 6.2 indicates increased binding between the GFP-Dsc1 and mCh-CortI at lower pH values. All P values were obtained by performing a Kruskal–Wallis test followed by the Wilcoxon–Mann–Whitney test (*P<0.05, **P <0.01, and ***P <0.001).

Throughout the high- and low-speed actin cosedimentation assays, GFP-Dsc1 was a relatively insoluble protein as the percent insoluble GFP-Dsc1 ranged from 20% to 40% depending on pH. Interestingly, mixing GFP-Dsc1 and mCh-CortI in high-speed cosedimentation assays caused a decrease in the insolubility of GFP-Dsc1 at most pH values. At a pH of 6.2, the GFP-Dsc1 insoluble fraction significantly decreased, indicating possible binding between GFP-Dsc1 and mCh-CortI (Fig. 5 B). Additionally, the solubility of Dsc1 decreases at lower pH values in low-speed cosedimentation assays, indicating these proteins are forming larger complexes (Fig. 5 C). Additionally, the imaging-based crosslinking and bundling assay indicated that actin crosslinking and bundling decreased when adding GFP-Dsc1 to the mCh-CortI and actin reaction at a pH of 6.2 (Fig. S6 C compared with Fig. S6 B). Taken together, we hypothesized that mCh-CortI and GFP-Dsc1 could bind to each other at a pH of 6.2 to create CK-like structures, which prevents these proteins from binding to and crosslinking/bundling actin (Fig. 5 A).

**Figure S9.**
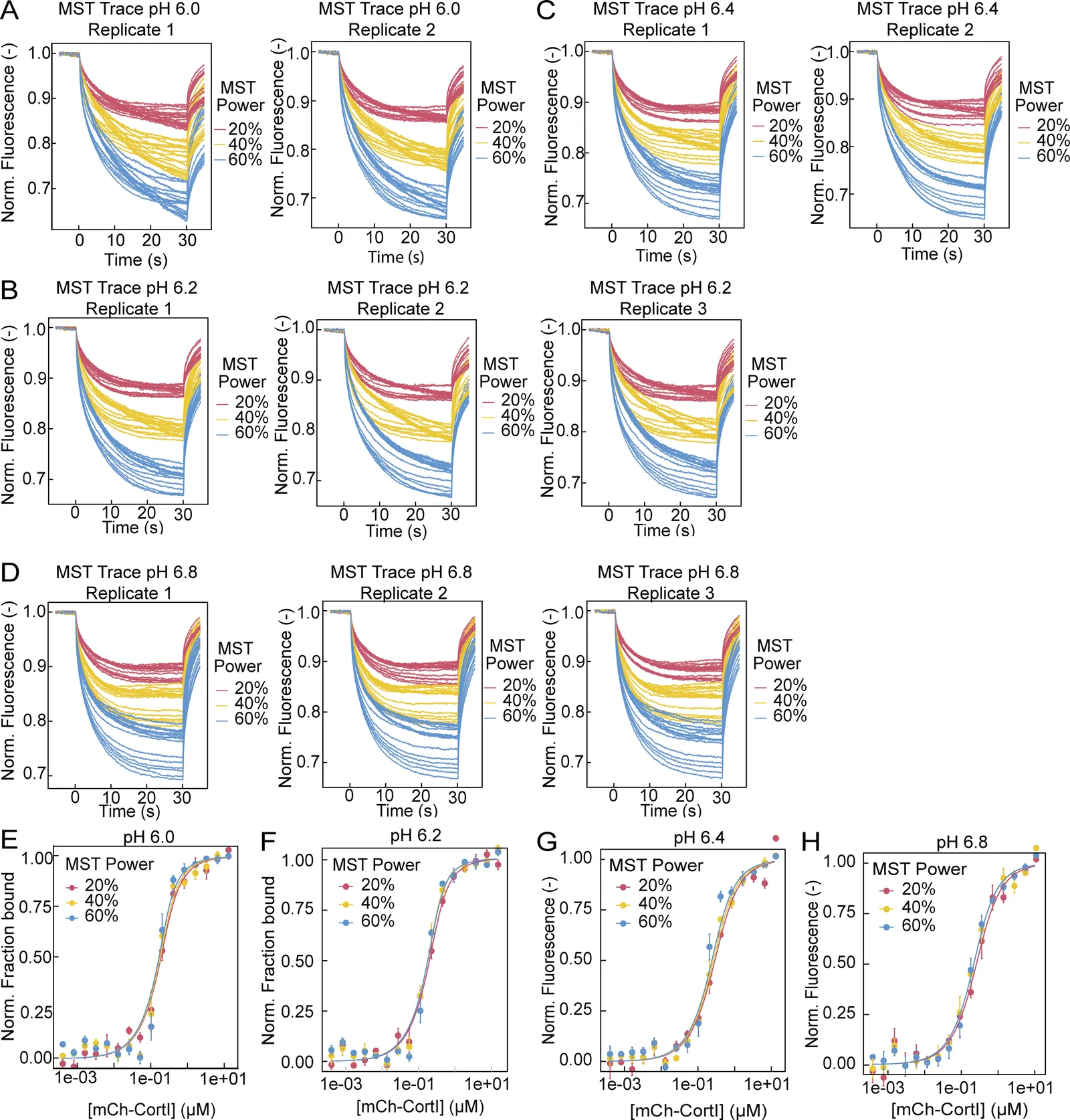
**Binding between mCh-CortI and GFP-Dsc1. (A–** **D** **)** MST trace for each replicate measuring mCh-CortI and GFP-Dsc1 binding at 20%, 40%, and 60% MST power levels for (A) pH 6.0 (third replicate shown in main text Fig. 4 C), (B) pH 6.2, (C) pH 6.4 (third replicate shown in main text Fig. 4 D), and (D) pH 6.8. **(E–H)** Normalized fraction bound of GFP-Dsc1 to mCh-CortI at different mCh-CortI concentrations at 20%, 40%, and 60% MST power levels at (E) pH 6.0, (F) pH 6.2, (G) pH 6.4, and (H) pH 6.8 (average of n = 3 per binding curve; bars represent the SEM).

Since CortI and Dsc1 are both part of the CKs, we wanted to test whether they directly bind to each other. Using microscale thermophoresis (MST), the binding between GFP-Dsc1 and mCh-CortI was tested at pH 6.0, 6.2, 6.4, and 6.8. The steep decrease in normalized fluorescence intensity indicates change in the thermophoretic mobility of GFP-Dsc1 upon binding to mCh-CortI as the bound complex moves faster out of the heated spot compared with the unbound molecule (Fig. 5, D and E; and Fig. S9, A–D). The MST power used did not significantly affect the binding curve created (Fig. S9, E–H). At all pH values, GFP-Dsc1 and mCh-CortI directly bound to each other; however, the strongest binding occurred at the lower pH values (6.0 and 6.2) (Fig. 5, F and G). At all pH values tested, CortI and Dsc1 bind to each other, likely forming CK-like structures.

### The cortical phase of the cell is slightly more acidic than the cytoplasmic phase

**Figure 6.**
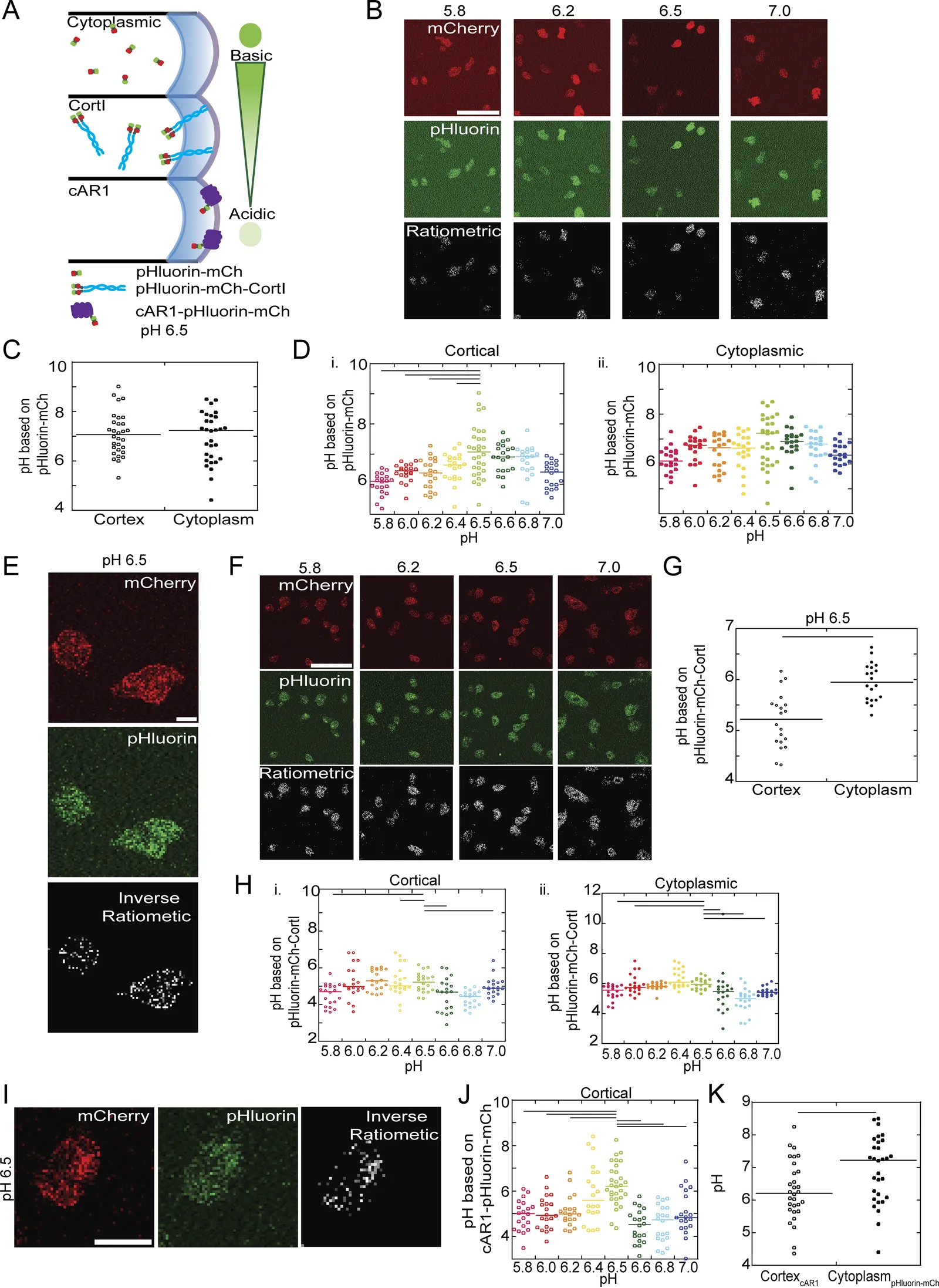
**Cortical phase of the cell is more acidic than the cytoplasmic phase of the cell. (A)** Graphic depiction of pHluorin-mCh constructs used in KAx3 cells to measure intracellular pH. **(B)** Representative images of KAx3::pHluorin-mCh:pDM181 cells in varying pH media (scale bar = 50 μm). **(C)** Quantification of subcellular pH when KAx3::pHluorin-mCh:pDM181 cells are incubated in pH 6.5 media, indicating no difference in pH between cortex and cytoplasmic phases. **(D)** Quantification of (i) cortical and (ii) cytoplasmic pH when KAx3::pHluorin-mCh:pDM181 cells are incubated in varying pH media. Increasing or decreasing the pH of media causes a decrease in pH of cell’s cortical and cytoplasmic phases. pH 6.5 data are represented again. **(E)** Representative images of KAx3::pHluorin-mCh-CortI:pDM181 cells in pH 6.5 media (scale bar = 5 μm). When pHluorin-mCh-CortI is excited with a 561-nm laser, CortI enrichment at the cortex is visible. However, when pHluorin-mCh-CortI is excited with a 488-nm laser, no significant visible difference in fluorescence intensity is observed, indicating that the cortex is more acidic. **(F)** Representative images of KAx3::pHluorin-mCh-CortI:pDM181 cells in varying pH media (scale bar = 50 μm). **(G)** Quantification of subcellular pH when KAx3::pHluorin-mCh-CortI:pDM181 cells are incubated in pH 6.5 media, indicating a difference in pH between cortical and cytoplasmic phases. **(H)** Quantification of (i) cortical and (ii) cytoplasmic pH when KAx3::pHluorin-mCh-CortI:pDM181 cells are incubated in varying pH media. Increasing or decreasing the pH of media causes a decrease in pH of the cell’s cortical and cytoplasmic phases. pH 6.5 data are presented again for direct side-by-side comparison. **(I)** Representative images of KAx3::cAR1-pHluorin-mCh:pDM181 cells in pH 6.5 media (scale bar = 5 μm). **(J)** Quantification of cortical pH when KAx3::cAR1-pHluorin-mCh:pDM181 cells are incubated in varying pH media. Increasing or decreasing the pH of media causes a decrease in pH of cell’s cortical phase. **(K)** Quantified difference in pH between cortical and cytoplasmic phases when comparing cytoplasmic expression of pHluorin-mCh to cAR1-pHluorin-mCh at a pH of 6.5 for side-by-side comparison; data are also presented earlier. All P values were obtained by performing a Kruskal–Wallis test followed by the Wilcoxon–Mann–Whitney test with bars indicating a P value <0.05.

**Figure S10.**
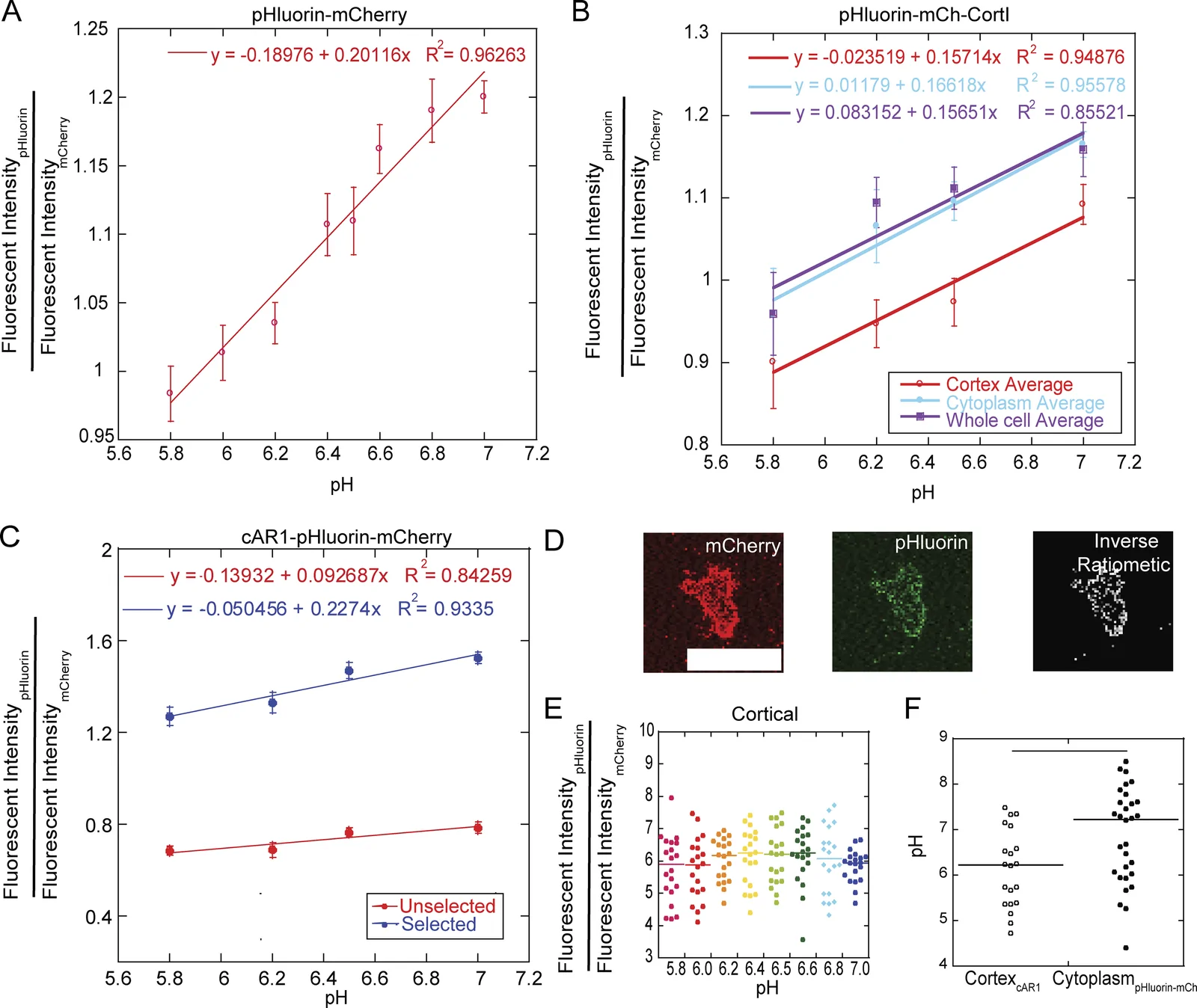
**Calibration curve of pH using pHluorin-based probes and subcellular pH measurements of selected KAx3::cAR1-pHluorin-mCh:pDM181 cells. (A)** Calibration curve used to relate fluorescence intensity of pHluorin to pH in KAx3::pHluorin-mCh:pDM181 cells (average of n = 20 per calibration curve; bars represent the SEM). **(B)** Calibration curve used to relate fluorescence intensity of pHluorin to pH in KAx3::pHluorin-mCh-CortI:pDM181 cells using either whole cell, cortical phase, or cytoplasmic phase of the cell (average of n = 20 per calibration curve; bars represent the SEM). **(C)** Calibration curve used to relate fluorescence intensity of pHluorin to pH in KAx3::cAR1-pHluorin-mCh:pDM181 cells (selected and unselected cells) (average of n = 20 per calibration curve; bars represent the SEM). **(D)** Representative images of selected KAx3::cAR1-pHluorin-mCh:pDM181 cells incubated in pH 6.5 media (top: scale bar = 20 μm). **(E)** Quantification of cortical pH for selected KAx3::cAR1-pHluorin-mCh:pDM181 cells across varying pH values. **(F)** Quantified difference in pH between cortical and cytoplasmic phases when comparing cytoplasmic expression of pHluorin-mCh to selected cAR1-pHluorin-mCh at a pH of 6.5 for side-by-side comparison; Cortex_cAR1_ data are represented previously in Fig. S10 E, and Cytoplasm_pHluorin-mCh_ data are represented previously in Fig. 6. All P values were obtained by performing a Kruskal–Wallis test followed by the Wilcoxon–Mann–Whitney test with bars indicating P value <0.05.

Overall, these data have led to the hypothesis that CKs form in the cytoplasm where the pH is higher and disassemble to associate with actin at the cortex where the pH is lower. To test this, the internal pH of the cell was measured using three different pH-sensitive fluorescent probes based on pHluorin (Fig. 6 A)—free cytoplasmic pHluorin (pHluorin-mCh), one linked to CortI (pHluorin-mCh-CortI), and one linked to an integral membrane protein, cAR1 (cAR1-pHluorin-mCh). The fluorescence intensity of the cortex and cytoplasmic regions of the cell was measured, and the pH was determined based on a standard curve (Fig. S10, A–C). In KAx3::pHluorin-mCh:pDM181 cells, no difference is observed between the cortex and cytoplasmic regions (Fig. 6, B and C); however, the expression of the protein is not localized in the cell, and we are unable to clearly differentiate the cortex and cytoplasm. Therefore, the pH of these cells is likely representative of the cytoplasmic pH. Changing the pH of the media causes a decrease in the pH of the cell cortex and cytoplasm (Fig. 6 D), which could explain why CortI and MyoII appear to be crosslinking and bundling actin through the cytoplasm when the pH of the media changes (Fig. 1, B–E).

To better differentiate the cortical and cytoplasmic phases of the cells, pHluorin-mCh-CortI:pDM181 was transformed into KAx3 cells (Fig. 6, E and F). In the mCh channel, CortI has a slight cortical enrichment in the cell; however, the fluorescence intensity of pHluorin appears similar between the cortical and the cytoplasmic phases at a pH of 6.5 (Fig. 6 E). When calculating the pH of the cytoplasmic and cortical phases, the cytoplasmic phase is more basic compared with the cortical phase (Fig. 6 G). Once again, increasing or decreasing the pH of the media increases the acidity of both the cortical and cytoplasmic phases of these cells (Fig. 6 H). However, it is important to note that the pH of the cortical and cytoplasmic regions of the KAx3::pHluorin-mCh-CortI:pDM181 cells were measured based on the calibration curve that utilized the cytoplasmic phase of the cell. When creating the calibration curve for these cells, the fluorescence intensity of the pHluorin and mCh channel was taken for the cortex, cytoplasm, and whole cells. All calibration curves gave different pH measurements, but the whole cell and cytoplasmic phase calibration curve were similar (Fig. S10 B). We chose to use the cytoplasmic calibration curves because we hypothesize that the cell has an innate mechanism in which the cortical phase is more acidic; therefore, the cytoplasmic region should be the more accurate pH measurement. Additionally, the cytoplasmic region of pHluorin-mCh and pHluorin-mCh-CortI expression cells is slightly different; however, even small changes to the method of pH measurement can lead to difference in the pH measured (Furukawa et al., 1988). Additionally, the KAx3::pHluorin-mCh-CortI:pDM181 cells have some punctate structures throughout the cytoplasm; therefore, it is possible that the pHluorin-mCh-CortI protein is being sequestered into lysosomes. Since lysosomes are typically more acidic, the overall pH measurements could be slightly reduced. Overall, these results indicate that the cortical region is more acidic than the cytoplasmic region, indicating that pH differences between the cortex and cytoplasm may serve as a mechanism through which these two phases are separated; however, these data only indicate a correlative effect and do not prove causality.

To validate this hypothesis, cAR1-pHluorin-mCh was used to measure the pH underneath the membrane (Fig. 6 I). cAR1 is a G protein–coupled receptor, which localizes to the membrane region. The pHluorin-mCh was attached on the inner cell side of the cAR1 protein (Fig. 6 A). The cortical region of the cell measured by cAR1-pHluorin-mCh was more acidic by increasing or decreasing pH similar to both pHluorin-mCh and pHluorin-mCh-CortI (Fig. 6 J). Interestingly, the cortical pH measured by cAR-pHluorin-mCh and the cytoplasmic pH measured by pHluorin-mCh indicate that the cortical phase of the cell is slightly more acidic than the cytoplasmic phase. It is important to note that the cytoplasmic pH measurement and the cortex pH measurement are not directly from the same cell. Furthermore, cAR1-pHluorin-mCh is localized to the membrane in our transformed cells; however, a bright area of fluorescence appears within the center of the cell (Fig. 6 I), which is likely the endoplasmic reticulum or Golgi apparatus. Since cAR1-pHluorin-mCh should localize completely to the membrane, we attempted to select for cells that had stronger cortical localization (Fig. S10 D). When measuring the cortical pH of the selected KAx3::cAR1-pHluorin-mCh:pDM181 cells across a range of pH values, increasing or decreasing the external pH of the media trended toward a slight increase in acidity, but the difference was not statistically significant (Fig. S10 E). However, the cortical pH measured by the selected cAR1-pHluorin-mCh cells and the cytoplasmic pH measured by pHluorin-mCh cells once again indicate that the cortical phase of the cell is slightly more acidic than the cytoplasmic phase (Fig. S10 F). Overall, these data suggest that the cell’s cortical phase is more acidic than the cytoplasmic phase, which could be one of many regulators that facilitate the transition between actin cortex construction and CK formation. Again, these data do not prove causality and only a correlative effect.

### Knockout of NHE1 changes the pH-dependent cortical localization, but not insolubility, of CortI and MyoII

**Figure 7.**
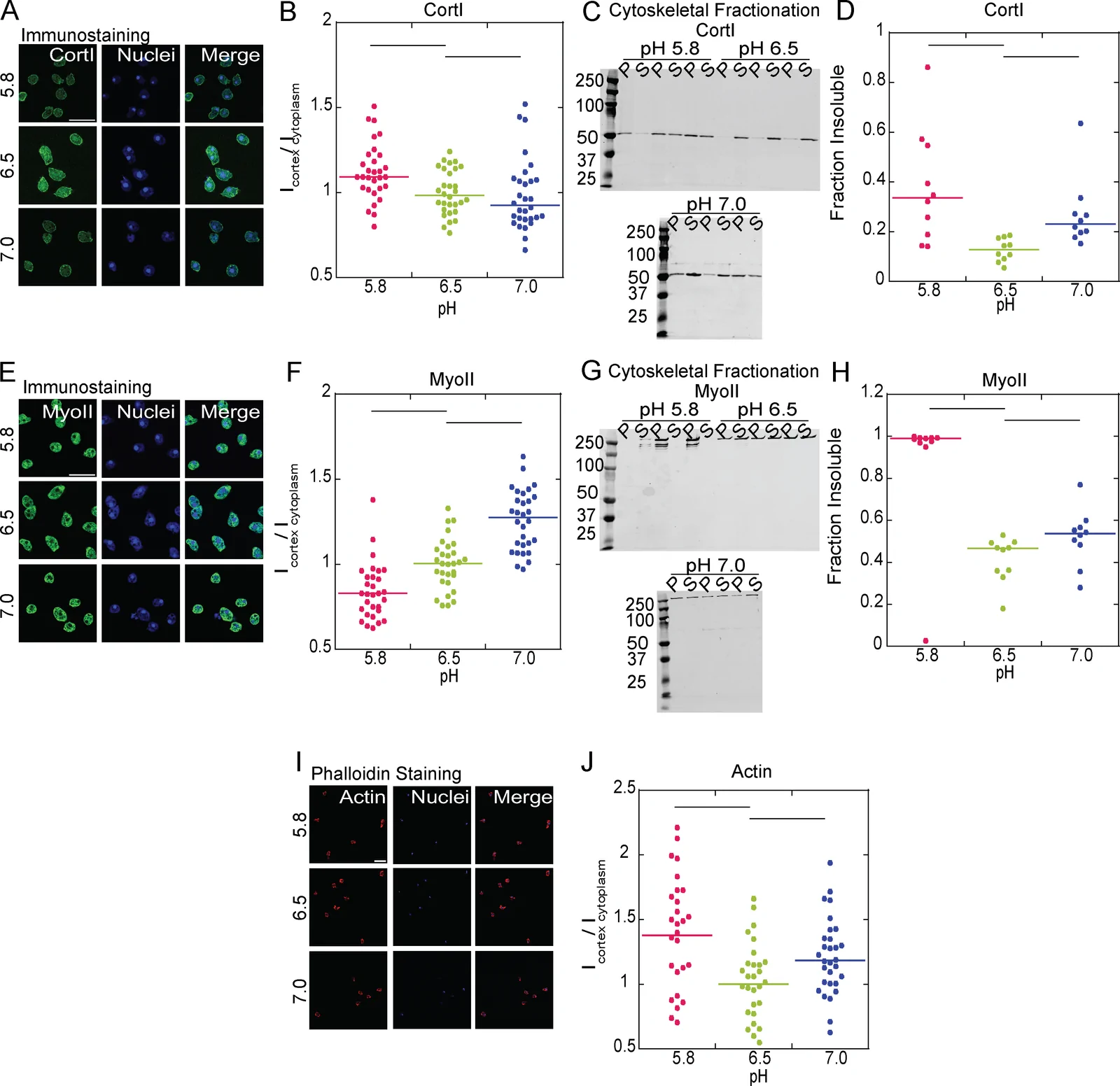
**Effects of pH on cortical localization of CortI, MyoII, and actin in *nhe1*** ^***−***^ **cells. (A)** Representative images of immunofluorescence imaging of CortI (green) and nuclei (blue) when incubated in media of varying pH values for 1 h. Images were collected on a confocal microscope (scale bar = 20 μm). **(B)** Quantification of CortI cortical localization by measuring the ratio of average fluorescence intensity at the cortex (I_Cortex_) to the cytoplasm (I_Cytoplasm_). Data were normalized to 6.5 by the day the slide was stained to account for any variation in day-to-day staining. **(C)** Representative western blot using mouse α-CortI for a cytoskeletal fractionation assay after cells had been incubated in varying pH media for 1 h. **(D)** Quantification of insoluble fraction of CortI by measuring integrated density of pellet and supernatant fractions. **(E)** Representative images of immunofluorescence imaging of MyoII (green) and nuclei (blue) when incubated in media of varying pH values for 1 h. Images were collected on a confocal microscope (scale bar = 20 μm). **(F)** Quantification of MyoII cortical localization by measuring the ratio of average fluorescence intensity at the cortex (I_Cortex_) to the cytoplasm (I_Cytoplasm_). Data were normalized to 6.5 by the day the slide was stained to account for any variation in day-to-day staining. **(G)** Representative western blot using mouse α-MyoII for a cytoskeletal fractionation assay after cells had been incubated in varying pH media for 1 h. **(H)** Quantification of insoluble fraction of MyoII by measuring integrated density of pellet and supernatant fractions. **(I)** Representative images of rhodamine-phalloidin–stained actin (red) and nuclei (blue) when incubated in media of varying pH values for 1 h. Images were collected on a confocal microscope (scale bar = 20 μm). **(J)** Quantification of actin cortical localization by measuring the ratio of average fluorescence intensity at the cortex (I_Cortex_) to the cytoplasm (I_Cytoplasm_). Data were normalized to 6.5 by the day the slide was stained to account for any variation in day-to-day staining. All P values were obtained by performing a Kruskal–Wallis test followed by the Wilcoxon–Mann–Whitney test with bars indicating a P value <0.05. Source data are available for this figure: SourceData F7.

NHE1 is a protein pump that regulates intracellular pH; therefore, NHE1 could affect cytoskeletal protein localization and solubility when cells are incubated in varying pH media. Specifically, *nhe1*^*−*^ cells were incubated in pH 5.8, 6.5, or 7.0 media and were immunostained for CortI (Fig. 7 A). In standard media (pH 6.5), CortI strongly localized to the cortex in *nhe1*^*−*^ cells. Increasing the pH of the media resulted in a decrease in CortI cortical localization (similar to WT cells); however, decreasing the pH of the media resulted in an increase in CortI cortical localization (Fig. 7, A and B). When staining for MyoII, increasing the pH of the media increased the cortical localization of MyoII (following the same trend as WT cells); however, decreasing the pH of the media resulted in a decrease in MyoII cortical localization (Fig. 7, E and F). When staining actin with rhodamine-phalloidin, increasing or decreasing the pH of the media results in an increase in cortical localization of actin, following the same trend as WT cells (Fig. 7, I and J). Therefore, NHE1 likely contributes to the pH-dependent localization of CortI and MyoII.

When performing cytoskeletal fractionation assays on the *nhe1*^*−*^ cells, increasing or decreasing the pH of the external media increased the insoluble fraction of both CortI and MyoII following the same trend as the WT cells (Fig. 7, C, D, G, and H). Therefore, NHE1 likely does not affect CortI or MyoII ability to interact with actin.

## Discussion

**Figure 8.**
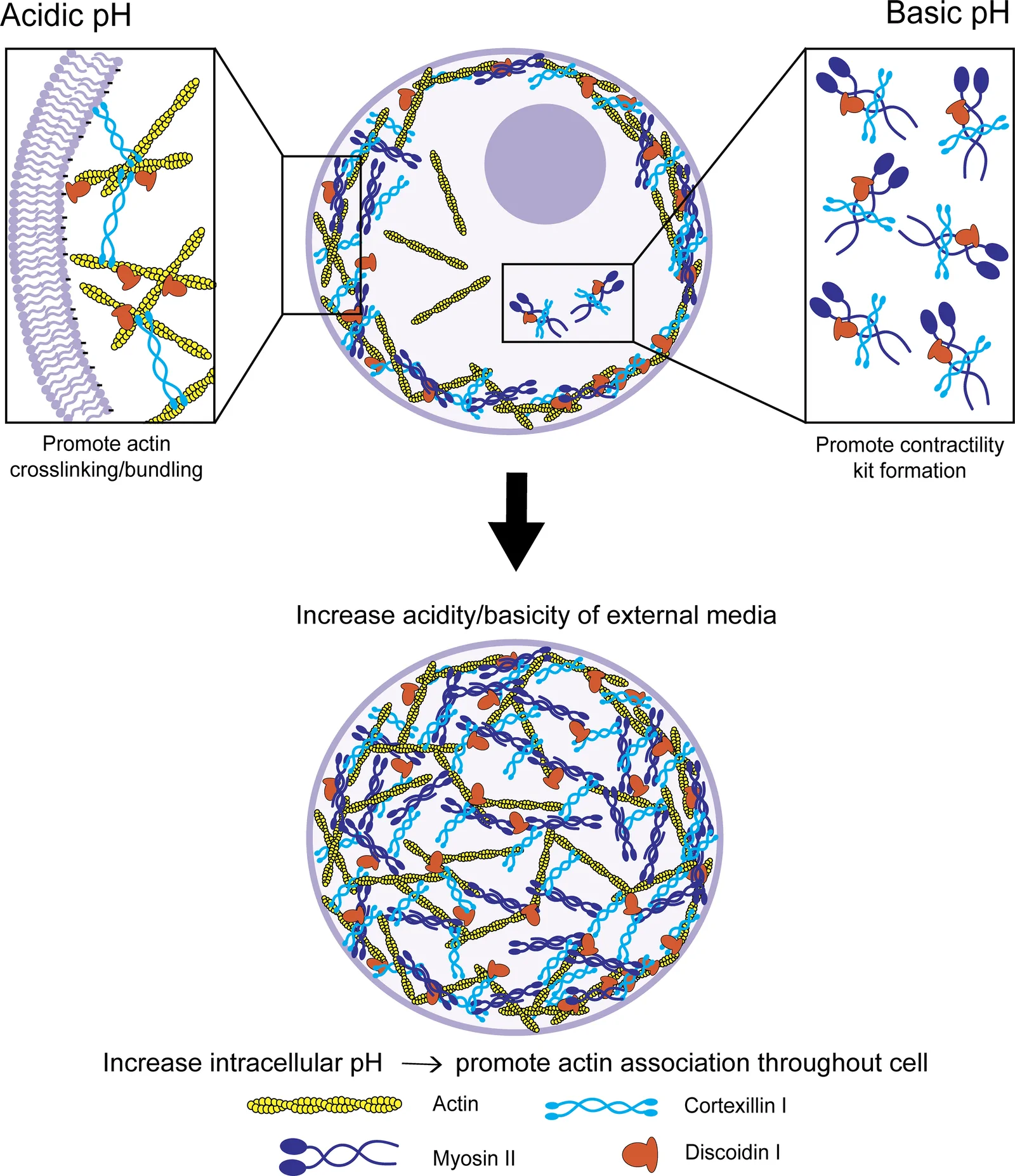
**Localized pH differential causes a differentiation between cortex and cytoplasmic phases in cells.** Filamentous actin is present throughout the cells; however, the actin cortex forms directly beneath the plasma membrane creating two separate phases (the cortical and cytoplasmic phases). In the cortical phase of a cell, the pH is slightly more acidic, leading to increased CortI- and Dsc1-mediated actin crosslinking and bundling to form the cortex. Since many phospholipids have negatively charged head groups, the lipids could attract H^+^ ions to the membrane, causing a slightly more acidic environment. In the cytoplasmic phase of the cell, the pH is slightly more basic, thereby causing an increase in CK formation. Increasing or decreasing the pH of the external media causes a decrease in the overall intracellular pH. The lower intracellular pH promotes actin cortex formation throughout the cells, which causes a loss in the cortex–cytoplasm phase separation.

Using both *in vivo* cellular assays and *in vitro* biochemical assays, we found that a pH differential between the cortical and cytoplasmic regions of the cell is a potential contributor to the regulation of cortex assembly and CK formation (Fig. 8). Using multiple pH-dependent fluorescent probes, we found that the cortical phase of the cell is slightly more acidic than the cytoplasmic phase. The increased acidity in this region likely promotes actin cortex formation. In the cytoplasmic phase of the cell, the pH is slightly more basic, which likely favors CK formation. Changing the external pH of the media increases the acidity of the cell (Fig. 6, D, H, and J; and Fig. S10 F). The increase in acidity of the cell overall could potentially promote actin cortex–like structures throughout the whole cell, leading to a redistribution of CortI, MyoII, and actin seen when changing the extracellular pH (Fig. 1, B, C, F, G, J, and K). It is important to note that while this conclusion is based on a large amount of *in vivo* and *in vitro* reconstitution data, fully confirming causality *in vivo* is nontrivial.

Although our data indicate that a pH differential helps mediate the transition between cortex formation and cytoskeletal protein condensate formation, other factors and ionic gradients likely exist that further facilitate the cortex–cytoplasmic phase separation. Specifically, Ca^2+^ significantly affects actin–crosslinker interactions (Yumura and Kitanishi-Yumura, 1993) and could further contribute to the separation between cortex and cytoplasm. Furthermore, other cytoskeletal protein regulators (Rho and Rac proteins) could be pH-sensitive themselves and further contribute to the changes in cytoskeletal interactions and localization we observed in this study.

Additionally, we confirmed that CortI is a pH-dependent actin crosslinker and discovered that Dsc1 itself is a weak actin crosslinker, with both proteins having increased interactions with actin at lower pH values. Previously, the binding affinity of CortI for actin was reported to be pH-independent (Faix et al., 1996); however, others have reported that CortI crosslinking and bundling is pH-dependent (Schmoller et al., 2012). Dsc1 affects CortI’s ability to interact with actin via direct binding to CortI at certain pH values. In our *in vitro* assay, we hypothesize that actin cortex–like structures form because CortI and Dsc1 will both bind to and crosslink/bundle actin together at lower pH values (5.8 and 6.0) despite CortI-Dsc1’s binding affinity for each other. As the pH increases, CK-like structures form as the affinity between CortI and Dsc1 outweighs the affinity of these proteins for actin. The binding affinity of these proteins for actin was determined by fitting a binding curve to a quadratic equation with the assumption of each protein having a single actin binding site. However, CortI has multiple actin binding sites, with each actin binding site having a slightly different affinity for actin (Stock et al., 1999). Further research should be conducted to determine how each actin binding site is affected by pH.

Moreover, Dsc1 has no known actin binding site, but crosslinks and bundles actin at lower pH values, indicating that Dsc1 has at least two distinct actin binding sites or forms multimers, allowing it to crosslink actin filaments. Structurally, Dsc1 has two domains: an N-terminal discoidin domain and a C-terminal lectin binding domain (Mathieu et al., 2010). We verified that actin binding and actin crosslinking and bundling are not affected by the addition of GalNAc, suggesting either that the lectin binding site does not bind to actin or that binding to GalNAc does not inhibit its ability to interact with actin. Furthermore, Dsc1 has been found to exist as a monomer, trimer, and tetramer depending on localization (Breuer and Siu, 1981; Fukuzawa and Ochiai, 1993). Based on immunofluorescence imaging using an antibody that only recognizes monomeric Dsc1, Dsc1 is present as a monomer in the cytoplasm but can take on multimeric forms when secreted outside of the cell. Determining the actin binding site on Dsc1 and the oligomeric state in which actin binds to Dsc1 would provide further insight into how Dsc1 itself is an actin crosslinker.

Throughout our past work, we determined that CortI and Dsc1 have intracellular interactions (Nguyen and Robinson, 2022). In *dsc1*^*−*^ cells, CortI cortical localization significantly decreases. Using *in vivo* fluorescence cross-correlation spectroscopy (FCCS), CortI and Dsc1 bound together; however, FCCS does not indicate direct binding. Additionally, the removal of Dsc1 from cells increased the apparent *in vivo* K_D_ (a qualified K_D_ to account for the measurement of the parameter in living cells) between CortI and MyoII in cells, indicating that Dsc1 was a mediator of CortI and MyoII interactions. In this study, we found Dsc1 and CortI directly bind to each other and interact in a pH-dependent manner, further confirming that Dsc1 is a component of the CK system. Understanding the binding structure between CortI and Dsc1 at varying pH values could elucidate CK formation.

Overall, these data indicate that cortex structures and CK formation are likely pH-dependent processes that are regulated by a pH differential between the cortical and cytoplasmic phases of the cell. On average, the cortical phase of the cell is slightly more acidic than the cytoplasmic phase in *Dictyostelium discoideum* cells. The mechanism by which the pH differential is maintained is currently unknown; however, the cell does have many ways in which it can regulate its internal pH. NHE1 is a membrane protein that pumps H^+^ ions out of the cell in exchange for Na^+^. NHE1 has been implicated in cell shape and mobility while also affecting actin-associated proteins’ interactions (Magalhaes et al., 2011; Martin et al., 2011; Patel and Barber, 2005). Here, we found that the removal of NHE1 can change the pH-dependent localization of CortI and MyoII. Further analysis into how NHE1 regulated other cytoskeletal protein localization and intracellular pH should be investigated. Additionally, other protein pumps in *Dictyostelium discoideum* can regulate and change the intracellular pH of the cell. The expression of the P-type H^+^-ATPase, *pat2*, increases rapidly when *Dictyostelium* cells are exposed to acidic environments to decrease the rate of acidification of the cell (Coukell et al., 1997), further suggesting that the cells can adapt to the change in external pH over time. Throughout the membrane in *Dictyostelium* cells, a high density of proton pumps congregates with increased localization throughout the contractile vacuole (Heuser et al., 1993). The presence of these protein pumps all throughout the cell membrane indicates that the cell typically removes excess H^+^ ions.

Furthermore, we found that increasing or decreasing extracellular pH led to a decrease in intracellular pH. Previous literature has indicated that internal cellular pH does not change when external pH is modified in *Dictyostelium discoideum* cells (Satre et al., 1986); however, the experiments measured how extracellular and intracellular pH changed as the *Dictyostelium discoideum* cells underwent differentiation and discovered that extracellular pH increased while intracellular pH remained the same. In our study, we directly changed the pH of the media and observed a change in intracellular pH. Decreasing the pH of the external media causes a decrease in overall intracellular pH likely because the cell is just taking on some of the free H^+^ ions that are present within the media. However, increasing the pH of the external media also decreased the overall intracellular pH. The cells may be able to survive with a cytosolic pH that is lower, but struggle with survival with a higher intracellular pH. Therefore, the cells might respond to higher pH by decreasing intracellular pH for survival. Over time, the cells likely return to “normal” intracellular pH as they adapt to the medium conditions. The addition of extracellular chemicals has previously been reported to decrease intracellular pH. For example, treating *Dictyostelium discoideum* cells with diethylstilbestrol (a chemical that induces stalk cell differentiation) causes a decrease in overall cytoplasmic pH (Furukawa et al., 1988) and promoted stalk formation. Understanding how changes to external pH lead to an increase in cytoplasmic acidity should be further studied.

Our data suggest that the cortical phase of the cell is on average more acidic than the cytoplasmic phase and that the mechanism in which the cell regulates intracellular pH is highly dependent on membrane-bound proteins; therefore, H^+^ ions must have an innate attraction to the membrane. Many of the phospholipids have a negatively charged head group. The large negatively charged surface could attract protons toward it through electrostatic forces, thereby increasing the local free proton concentration. Overall, the negatively charged phospholipid head groups are likely attracting protons to the membrane to increase cortical phase acidity, which, in turn, helps the cell to regulate its intracellular pH. Furthermore, biomolecular condensates are capable of sustaining pH gradients themselves (Ausserwöger et al., 2026). The actin cortex itself could be thought of as a phase-separated region of the cell and could possibly sustain the pH differential between the cortex and cytoplasm itself. Understanding how the cells can maintain this pH differential could provide insight into the mechanism that underpins overall cellular shape changes.

## Materials and methods

### *Dictyostelium* cell culture

All *Dictyostelium discoideum* strains used in this study are listed in Table S1. Cells were cultured in Hans’ enriched 1.5× HL-5 media with 8% FM Minimal (FM) media and penicillin and streptomycin. Cells were incubated at 22°C on nontissue culture-treated 10-cm petri dish if unspecified. When cells were grown in suspension culture, sterile 125-ml Erlenmeyer flasks with 10 ml of media were used. The cultures were grown at 22°C, rotating on a shaker at 180 rpm. The WT strain used was KAx3. To create transformants, KAx3 cells were electroporated using a Gene Pulser II electroporator and selected with 15 μg/ml of G418. For *Dictyostelium discoideum* transformations, two different plasmid backbones were used: pDM181 and pExp4. The pDM181 vector contains *Dictyostelium discoideum* act15 promoter, NeoR (G418R) drug resistance cassette, and a Dd origin of replication (ori). The pDM181 plasmid also contains a pUC ori and ampicillin resistance cassette for bacterial replication and selection. The pExp4 plasmid is derived from the pAT153L vector backbone and contains an act15 promoter for *Dictyostelium discoideum* Act6 neo resistance for selection. To select the KAx3::cAR1-pHluorin-mCh:pDM181 cells, the transformants were mixed with *Klebsiella aerogenes* and plated on SM5 plates. Individual *Dictyostelium discoideum* colonies were picked and grown up in 1.5× HL-5 media with 15 μg/ml of G418 and screened for strong cAR1 cortical expression. To create different pH media, Hans’ enriched 1.5× HL-5 media were made, and pH was set at stated pH before adding FM and penicillin and streptomycin. This process confirmed FM, penicillin, and streptomycin do not significantly change the pH of the media. It is important to note that the medium that was at a pH of 8.0 was significantly darker in color and had a noticeably different odor than all other media made. NHE1 knockout cells were obtained from dictyBase (DBS0236604) and have been previously described (Patel and Barber, 2005).

### Immunofluorescence imaging

250,000 cells were seeded on a 22 × 22 cm glass coverslip in a 6-well plate and incubated for 24 h. The medium of the cells was changed to a different media of the stated pH and incubated for 1 h. When staining with CortI or MyoII, the cells were fixed with −20°C methanol for 3 min followed by Fix Mix (4% PFA, 0.1% Triton X-100, 150 mM NaCl) for 5 min. Residual methanol and PFA were washed away with 1x PBST with 0.5% BSA. Cells were blocked in 5% BSA solution for 1 h and stained with 1:200 mouse α-CortI (Developmental Studies Hybridoma Bank (DSHB) 241-438-1; University of Iowa, Iowa City, IA, USA) or mouse α-MyoII (DSBH 56-396-5) overnight. 1:200 of goat α-mouse Alexa Fluor 488 (A-11001; Invitrogen) was used as the secondary antibody. Rhodamine-phalloidin–stained cells were fixed with Fix Mix, and residual PFA was washed away with 1xPBS. Cells were stained with 1:400 rhodamine-phalloidin (R415; Invitrogen) for 1 h. All cells were stained with 10 µg/ml Hoechst in PBST with 0.5% BSA. Stained coverslips were mounted onto slides using ProLong Gold Antifade Reagent (P36930; Invitrogen). MyoII and actin slides were imaged on Zeiss Light Scanning Confocal Microscope (LSM) 880 Airyscan FAST using a 63× (NA 1.4) PlanApo oil objective and the 880-Quasar GaAsp detector. KAx3 cells stained for CortI were imaged on a Zeiss LSM 700 equipped with a 40× (NA 1.30) Plan-Neofluar oil objective and a Bi-Akali detector. All images were taken using the Zeiss ZEN black software. All images were taken at room temperature. Images were analyzed using ImageJ/FIJI.

### Cytoskeletal fractionation assay

Media on cells were replaced with HL-5 of varying pH values and incubated for 1 h. For the latrunculin A–treated cells, 5 μM of latrunculin A was incubated with the cells for 30 min prior to fractionation (3973; Tocris Bioscience). 6 × 10^6^ cells were centrifuged at 400 × *g* for 5 min and washed with 1 ml pH-matched starvation buffer (50 mM MES, 0.2 M CaCl_2_, 2 mM MgCl_2_). Each sample was then separated into 2 samples to create technical replicates. Cells were centrifuged at 400 × *g* for 5 min and resuspended in 100 μl of lysis buffer (200 mM MES, 2.5 mM EGTA, 5 mM MgCl_2_, 1% Triton X-100, 1 mM ATP, 0.1 mM phenylmethanesulfonyl fluoride, 1 μg/ml aprotinin, 0.15 mM N-alpha-tosyl-L-lysyl-chloromethyl ketone, 5 μg/ml leupeptin, 1 μg/ml benzamidine, 2 μg/ml pepstatin A, 0.1 mg/ml tosyl-L-arginine methyl ester, and 80 mg/ml N-p-tosyl-L-phenylalanine chloromethyl ketone). The pH of the lysis buffer was matched to the pH of the media in which the cells were incubated. Cells were incubated on a rocker for 5 min and centrifuged at 10,000 × *g* for 7.5 min. Pellet and supernatant were separated, volume-normalized with 2× sample buffer, and boiled for 5 min before running on a 10% SDS-PAGE gel. The western blots were stained with 1:1,000 mouse α-CortI (DSHB 241-438-1; University of Iowa, Iowa City, IA, USA), mouse α-MyoII (DSBH 56-396-5), or mouse α-actin (DSHB 224-236-1) as primary antibodies. The secondary antibody used was IRDye 680LT Goat anti-Mouse IgG Secondary Antibody (LI-COR, 926-68020).

### Growth rate calculations

Starting with a cell concentration of 5.0 × 10^4^ cells, cells were seeded in 10 ml of HL-5 media on a non-tissue culture-treated 10-cm petri dish or in 10 ml of media in a 150-ml Erlenmeyer flask. All cells were grown at 22°C with the suspension cultures growing on a shaker set to 180 rpm. Approximately every 24 h, cell concentration was measured and recorded using a hemocytometer. For suspension-based curves, data were obtained and analyzed from the second cycle of suspension culture. Growth rates were found by plotting cell concentrations versus time. The exponential phase curve was fitted to a single exponential equation using KaleidaGraph. The following exponential growth equation was used to determine each growth curve:

$$n\left(t\right)=n_{0}e^{kt},$$

where

t = time

k = growth rate

n(t) = population at time

n_0_ = initial population concentration.

### Random migration assessment via live-cell imaging

Cells were grown on non–TC-treated 10-cm plates until 70% confluency was reached. Cells were seeded at a concentration of 2.0 × 10^5^ cells/500 μl into the wells of a 4-well chamber (Thermo Fisher Nunc Lab-Tek II Chambered 1.5 German Coverglass System 8 Chamber, Ref. No. 155382 PK) and allowed to adhere for an hour at 22°C. Cells were incubated for 1 h in HL-5 of varying pH values.

Time-lapse DIC imaging was performed using an Olympus IX81 inverted microscope equipped with a 40×/1.3 NA oil immersion objective and a Roper Systems CCD camera. Images were acquired every 10 s for 10 min at room temperature. All images were taken using MetaMorph software.

Cell migration was analyzed in Fiji (ImageJ, NIH). Individual cells were manually outlined in each frame of the time-lapse sequence using the freehand selection tool to generate regions of interest (ROIs). The centroid coordinates of each ROI were extracted and used to calculate migration speed, accumulated distance, net displacement, and directionality. Cell area and shape descriptors, including circularity, were also measured from the segmented ROIs.

### Protein purification

BL21 cells containing a pLysS plasmid (BLAREs) were transformed with pBiEX-1 plasmid (Novagen DNA, 71234-3; Merck-Millipore) containing either mCh-CortI or GFP-Dsc1. The expression of the protein was induced with IPTG, and cells were incubated at 16°C for 24 (GFP-Dsc1) to 48 (mCh-CortI) h. Cells were lysed through flash freezing and sonication and purified with polyethyleneimine and ammonium sulfate before being further purified using fast protein liquid chromatography. GFP-Dsc1 was purified using a Ni-NTA column, using Ni-NTA Superflow resin (30410; Qiagen), followed by UnoS1 (720-0021; Bio-Rad) and UnoQ1 (720-0001; Bio-Rad) columns. mCh-CortI was purified using a HiPrep 26/60 Sephacryl S300 HR (95055-810; VWR) column followed by a Ni-NTA column (30410; Qiagen) and concentrated using an UnoQ1 (720-0001; Bio-Rad) column. All recombinant proteins were dialyzed in 10 mM HEPES, pH 7.1, 20 mM NaCl, 1 mM EDTA, and 1 mM EGTA. Actin was purified from chicken skeletal muscle (Pardee and Spudich, 1982). Chicken skeletal muscle was ground in a meat grinder. The ground chicken was incubated with KCl extraction solution (0.1 M KCl and 0.15 M KHPO_4_ [pH 6.5]) for 10 min, bicarb extraction solution (0.05 M NaHCO_3_) for 5 min, 1 mM EDTA (pH 7.0) for 10 min, and 18 MΩ dH_2_O for 5 min. All steps were performed at 4°C. Between each solution, powder was filtered through cheesecloth. To obtain acetone powder, the powder was incubated with 25°C acetone for 10 min and filtered through cheesecloth. The acetone extraction was repeated another 4 times. The acetone powder was air-dried overnight before being collected and stored at −80°C. To purify actin from acetone powder, acetone powder was mixed at 4°C for 30 min in buffer G (2 mM Tris [pH 8.0], 0.2 mM NaATP, 0.2 mM CaCl_2_, 0.005% sodium azide, and 0.5 mM β-mercaptoethanol) and was filtered through cheesecloth twice. The mixture was centrifuged at 46,000 × *g* for 30 min to remove any insoluble protein. The supernatant was then incubated at 25°C for 1 h in 50 mM KCl, 2 mM MgCl_2_, and 1 mM ATP solution. The concentration of KCl was raised to 0.6 M and incubated for another 15 min at room temperature, causing the actin to polymerize. To collect the polymerized actin, the solution was centrifuged at 205,000 × *g* for 60 min. The pellet was resuspended in buffer G and incubated for 15 min on ice to depolymerize actin. The solution was dialyzed in buffer G overnight. The solution was then centrifuged at 95,000 × *g* for 25 min to remove any insoluble protein and flash-frozen for storage.

### Actin crosslinking and bundling imaging assay

G-actin was polymerized by incubating actin in 50 mM MES (pH based on experiment), 4 mM MgCl_2_, 25 mM KCl, 1 mM ATP, 1 mM DTT for 1 h at 22°C. Samples were mixed with Alexa Fluor 647-phalloidin (A22287; Invitrogen). 2 μl of sample was added to a 96-well plate where a glass coverslip was placed on top, and samples were sealed with vacuum grease. Samples were incubated for 1 h, and Z-stack images at nine locations were collected using Molecular Devices IXM High-Content Imager equipped with a 40× (NA 0.95) PlanApo objective (0.16–0.25 mm working distance, correction collar) and a high-resolution scientific CMOS camera. All images were taken at room temperature using MetaXpress software. To analyze the images, the pixel intensity standard deviation was measured for the Z-slice that was in focus using ImageJ.

### High- and low-speed actin cosedimentation assay

G-actin and other proteins were centrifuged at 100,000 × *g* for 25 min prior to experimentation to remove any insoluble protein. G-actin was polymerized by incubating actin in 50 mM MES (pH based on experiment), 4 mM MgCl_2_, 25 mM KCl, 1 mM ATP, 1 mM DTT for 1 h at 22°C. The purified proteins were mixed and incubated at 22°C for 1 h. To assess actin binding, samples were centrifuged at 100,000 × *g* for 25 min. To assess for actin crosslinking and bundling, samples were centrifuged at 10,000 × *g* for 25 min. Pellet and supernatant were separated, volume-normalized using 2× sample buffer, and boiled at 95°C for 5 min in preparation to run on 10% SDS-PAGE gels. To create the binding curves and calculate K_D_ between actin and other proteins, the curves were fit to the following equation:

$$\frac{\left(\left[A_{P}\right]+\left[B_{T}\right]+K_{D}\right)-\sqrt{{\left(\left[A_{T}\right]+\left[B_{T}\right]+K_{D}\right)}^{2}-4\cdot \left[A_{T}\right]\cdot \left[B_{T}\right]}}{2},$$

where

A_P_ = concentration of pelleted actin

A_T_ = total concentration of filamentous actin

B_T_ = total concentration of other protein (mCh-CortI or GFP-Dsc1).

### Microscale thermophoresis

Binding affinities between purified mCh-CortI and GFP-Dsc1 were measured using MST. GFP-Dsc1 was held at a constant concentration, while mCh-CortI was titrated in 16-point serial dilutions in 50 mM MES (pH based on experiment), 4 mM MgCl_2_, 25 mM KCl, 2 mM DTT, 0.05% Tween-20 for 15 min at 23°C. Samples were loaded into standard capillaries (MO-K022) and measured on a Monolith NT.115 at 23°C using MST power levels of 20%, 40%, and 60% and 5% excitation power. Normalized fluorescent values (F_norm_ = F_0_/F_1_) were plotted against ligand concentration to generate dose–response curves, and K_D_ values were obtained by nonlinear least squares fitting to a 1:1 binding model based on the law of mass action using MO Affinity Analysis 3 software.

### Internal pH Quantification (pHluorin)

Media on cells were replaced with HL-5 of varying pH values and incubated for 1 h. Cells used to create standard curves were incubated in varying pH media with 5 μM nigericin, free acid (N1495; Invitrogen). After 1 h, media were removed and replaced with low fluorescent media of the same pH (with standard curve cells still containing nigericin). The cells were imaged on a Zeiss LSM 780 using a 40× (NA 1.3) Plan-Neofluar oil objective and the GaAsp detector using 488-nm (pHluorin) and 561-nm (mCh) excitation wavelengths. The average intensity of the cell cortex and cell cytoplasm was measured at each wavelength. The values reported were the average intensity at 488 nm divided by the average intensity at 561 nm.

### Statistical analysis

Datasets were statistically analyzed using the Kruskal–Wallis test followed by an unpaired Mann–Whitney–Wilcoxon test. P values <0.05 were considered statistically significant and are denoted by a black bar between the two datasets being compared. The statistical tests were performed on KaleidaGraph using the statistical test functions provided.

### Online supplemental material

Supplemental Materials include Figs. S1, S2, S3, S4, S5, S6, S7, S8, S9, S10, and Table S1. Fig. S1 shows the effects of pH on cortical localization of CortI and MyoII in live cells. Fig. S2 shows Dsc1 cytoskeletal fractionation assay. Fig. S3 shows latrunculin A–treated cytoskeletal fractionation assays. Fig. S4 shows difference in growth rate when cells are incubated in different media. Fig. S5 shows protein purification products. Fig. S6 shows high-throughput imaging assay to assess actin crosslinking and bundling by CortI and/or Dsc1 at varying pH values. Fig. S7 shows low-speed actin sedimentation assay curves at varying pH values. Fig. S8 shows binding between actin and mCh-CortI or GFP-Dsc1 at varying pH values. Fig. S9 shows binding between mCh-CortI and GFP-Dsc1. Fig. S10 shows calibration curve of pH using pHluorin-based probes and subcellular pH measurements of selected KAx3::cAR1-pHluorin-mCh:pDM181 cells. Table S1 shows a list of *Dictyostelium discoideum* strains used throughout this study.

## Supplementary Material

Table S1 shows a list of *Dictyostelium discoideum* strains used throughout this study.

SourceData F1 is the source file for Fig. 1.

SourceData F3 is the source file for Fig. 3.

SourceData F4 is the source file for Fig. 4.

SourceData F7 is the source file for Fig. 7.

SourceData FS2 is the source file for Fig. S2.

SourceData FS3 is the source file for Fig. S3.

SourceData FS5 is the source file for Fig. S5.

## Data Availability

All datasets are contained in the paper.
