# Supplementary material for: The effects of pH on cortex and condensate formation
Source: J Cell Biol. 2026 Oct 16;225(12):e202603108. doi: 10.1083/jcb.202603108 (PMC13674344; doi:10.1083/jcb.202603108)
Supplement: Supplementary file 2 [file jcb_202603108_sourcedataf3.pdf]

Low-Speed Actin Co-Sedimentation Assay pH 6.0

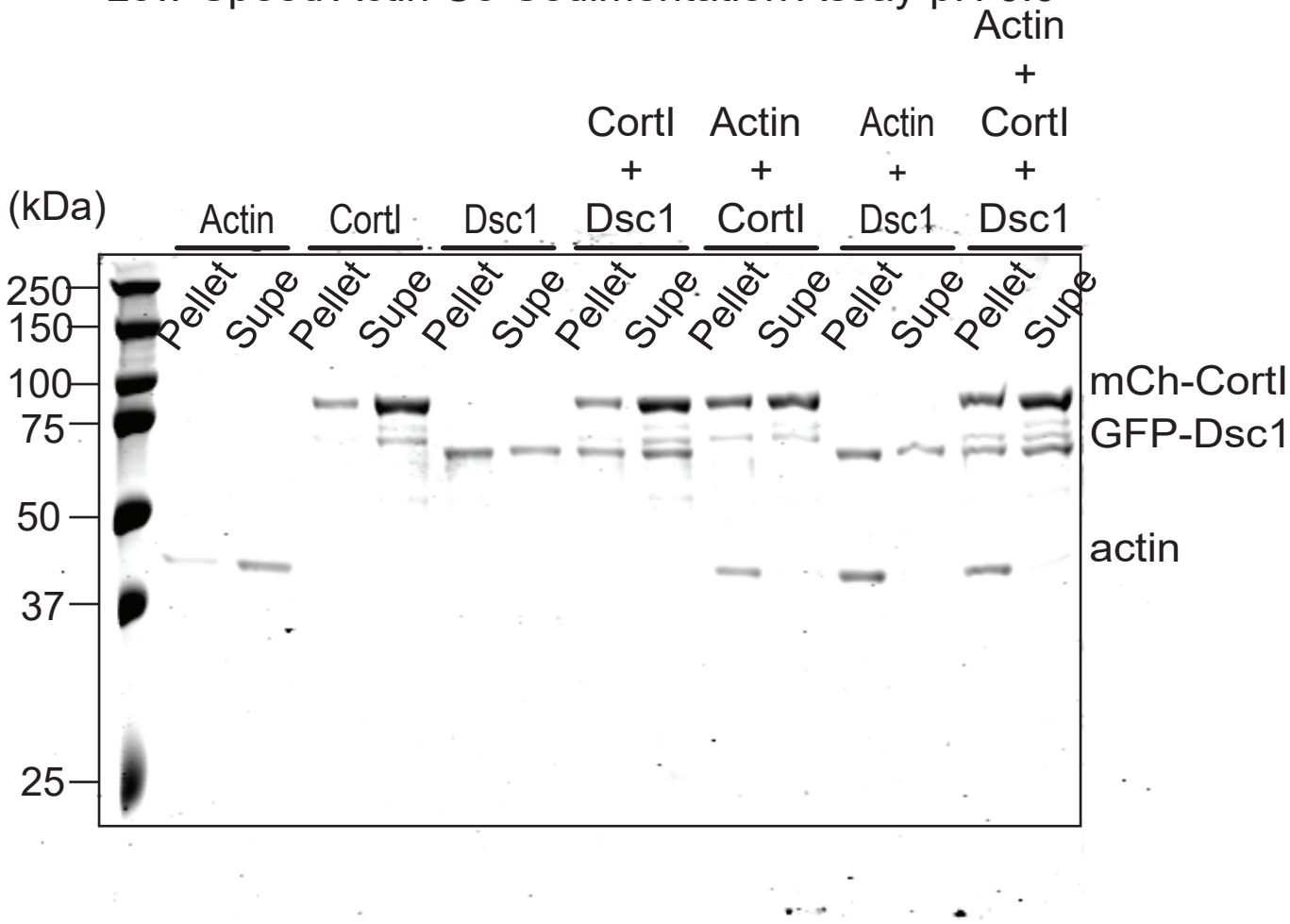

Coomassie Blue Stain

Low-Speed Actin Co-Sedimentation Assay pH 6.8

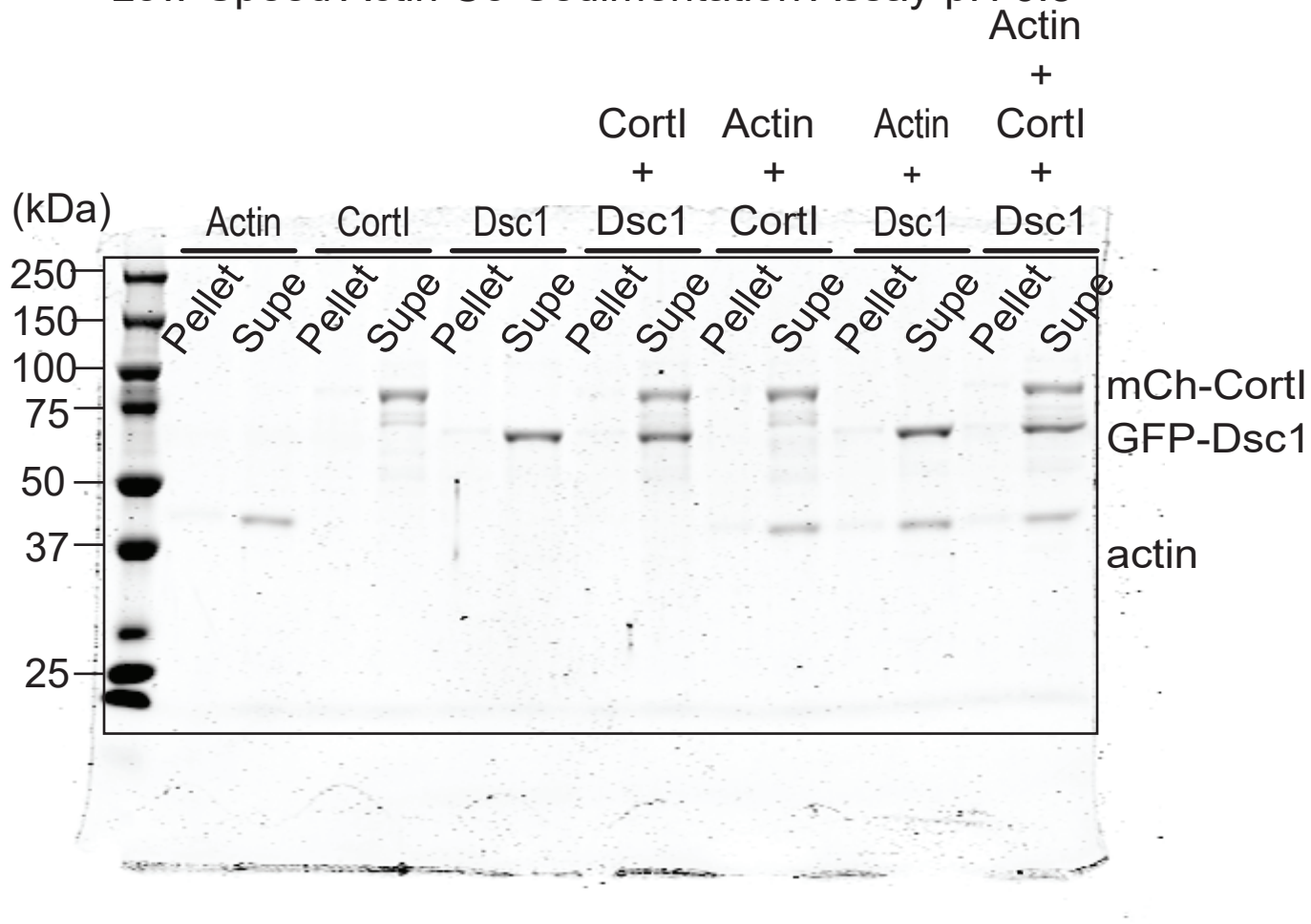

Coomassie Blue Stain
