# Supplementary material for: The effects of pH on cortex and condensate formation
Source: J Cell Biol. 2026 Oct 16;225(12):e202603108. doi: 10.1083/jcb.202603108 (PMC13674344; doi:10.1083/jcb.202603108)
Supplement: Supplementary file 4 [file jcb_202603108_sourcedataf7.pdf]

Cytoskeletal Fractionation
CortI

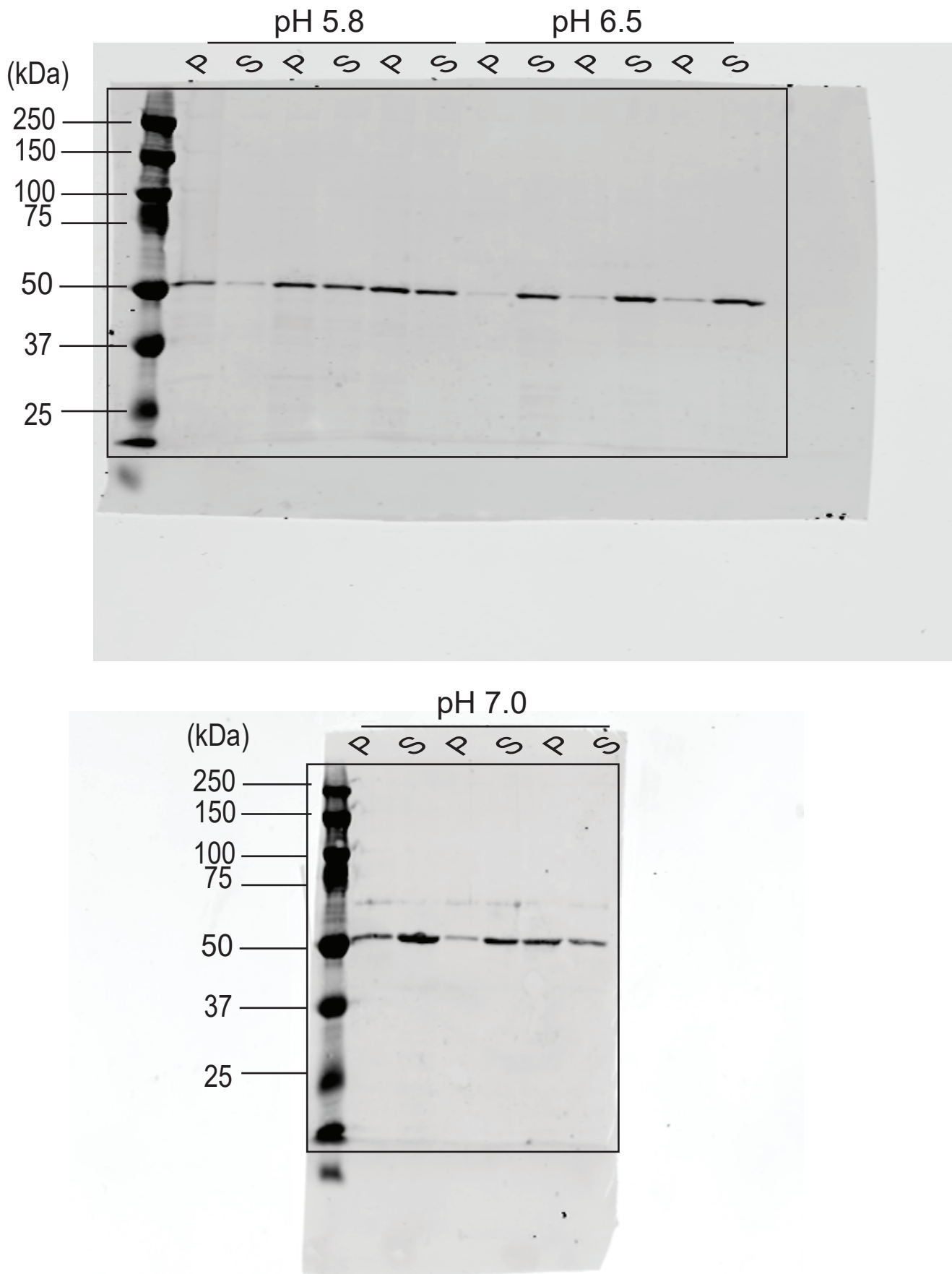

1° Antibody: Mouse anti-CortI
2° Antibody: Goat anti-Mouse-680

Cytoskeletal Fractionation
MyoII

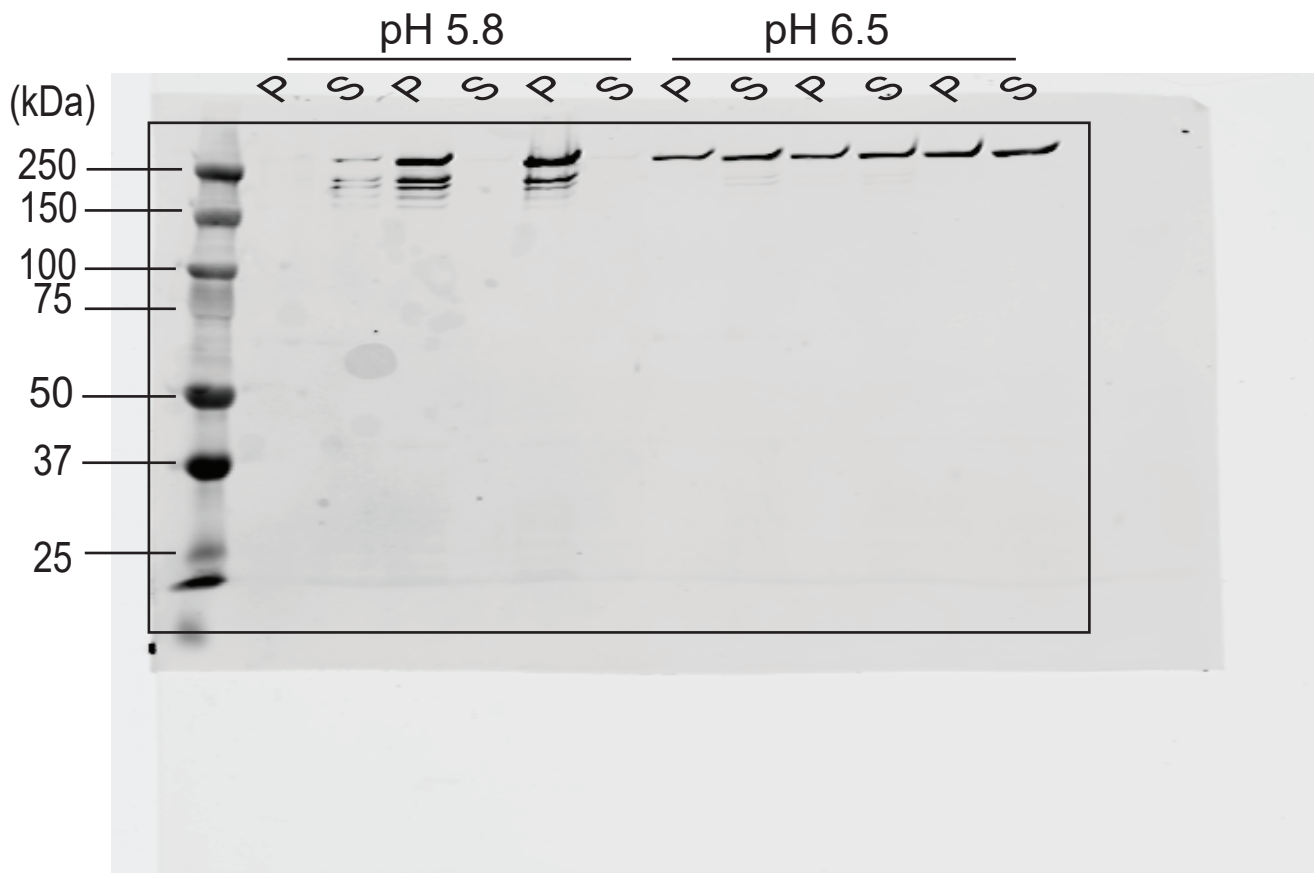

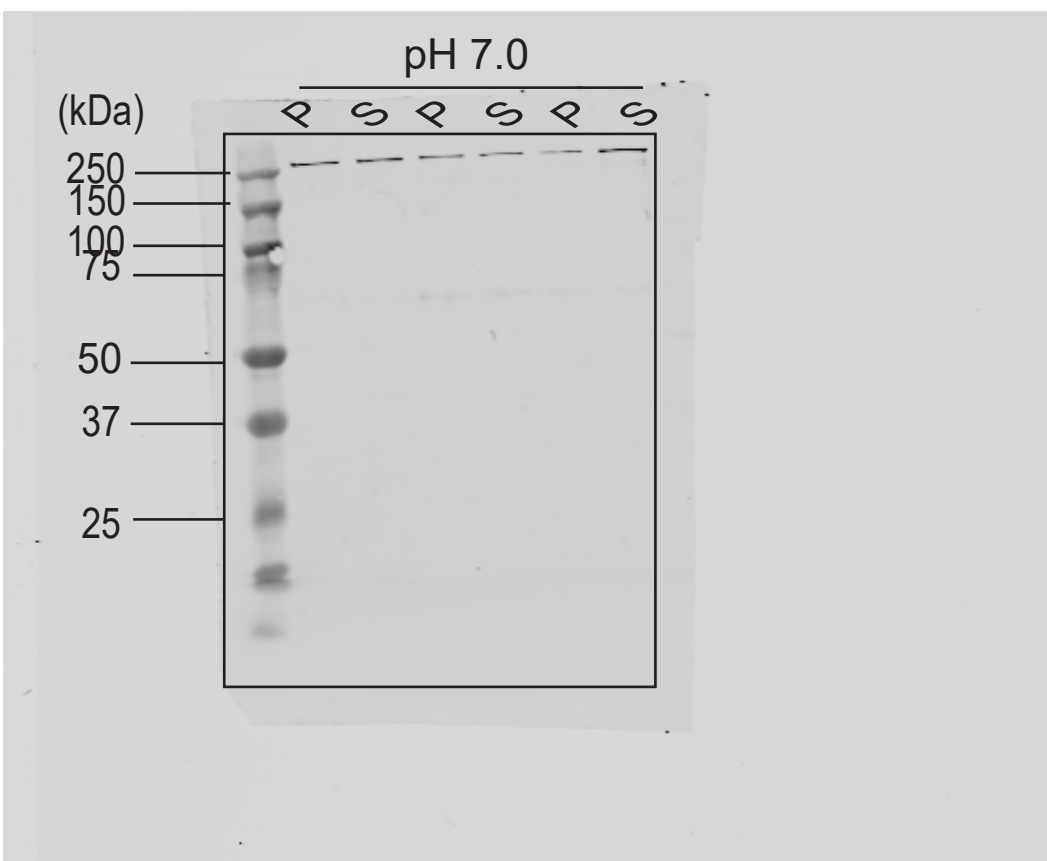

1° Antibody: Mouse anti-MyoII
2° Antibody: Goat anti-Mouse-680
