# Supplementary material for: The effects of pH on cortex and condensate formation
Source: J Cell Biol. 2026 Oct 16;225(12):e202603108. doi: 10.1083/jcb.202603108 (PMC13674344; doi:10.1083/jcb.202603108)
Supplement: Supplementary file 5 [file jcb_202603108_sourcedatafs2.pdf]

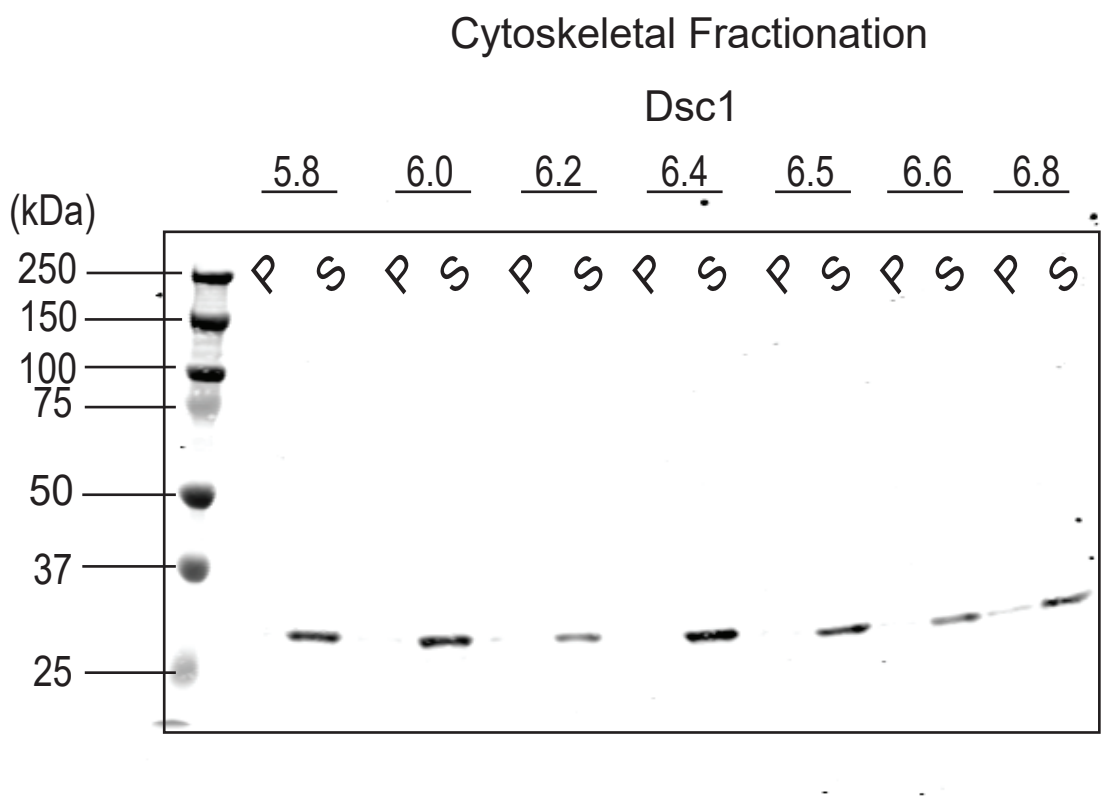

1° Antibody: Mouse anti-Dsc1
2° Antibody: Goat anti-Mouse-680
