# Supplementary material for: The effects of pH on cortex and condensate formation
Source: J Cell Biol. 2026 Oct 16;225(12):e202603108. doi: 10.1083/jcb.202603108 (PMC13674344; doi:10.1083/jcb.202603108)
Supplement: Supplementary file 7 [file jcb_202603108_sourcedatafs5.pdf]

Actin

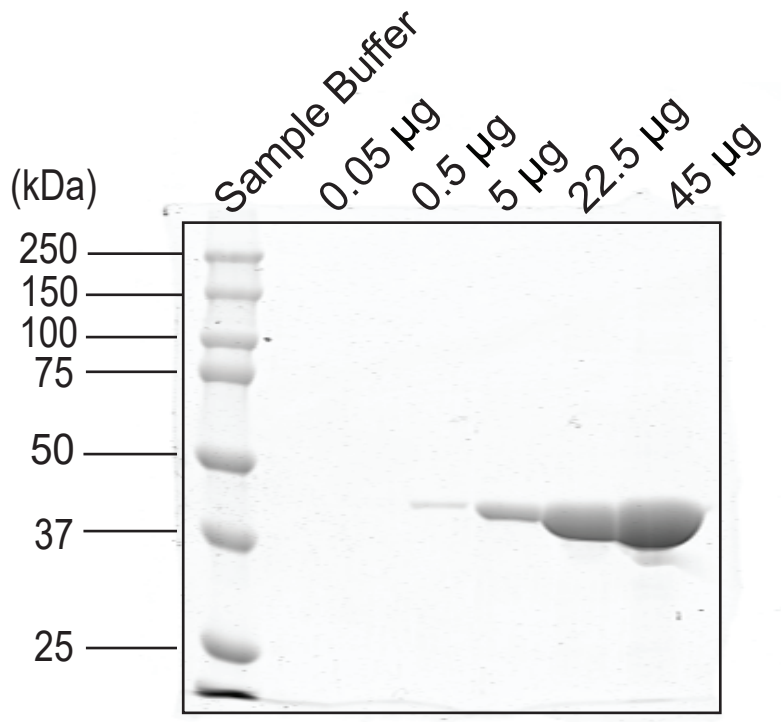

Coomassie Blue Stain

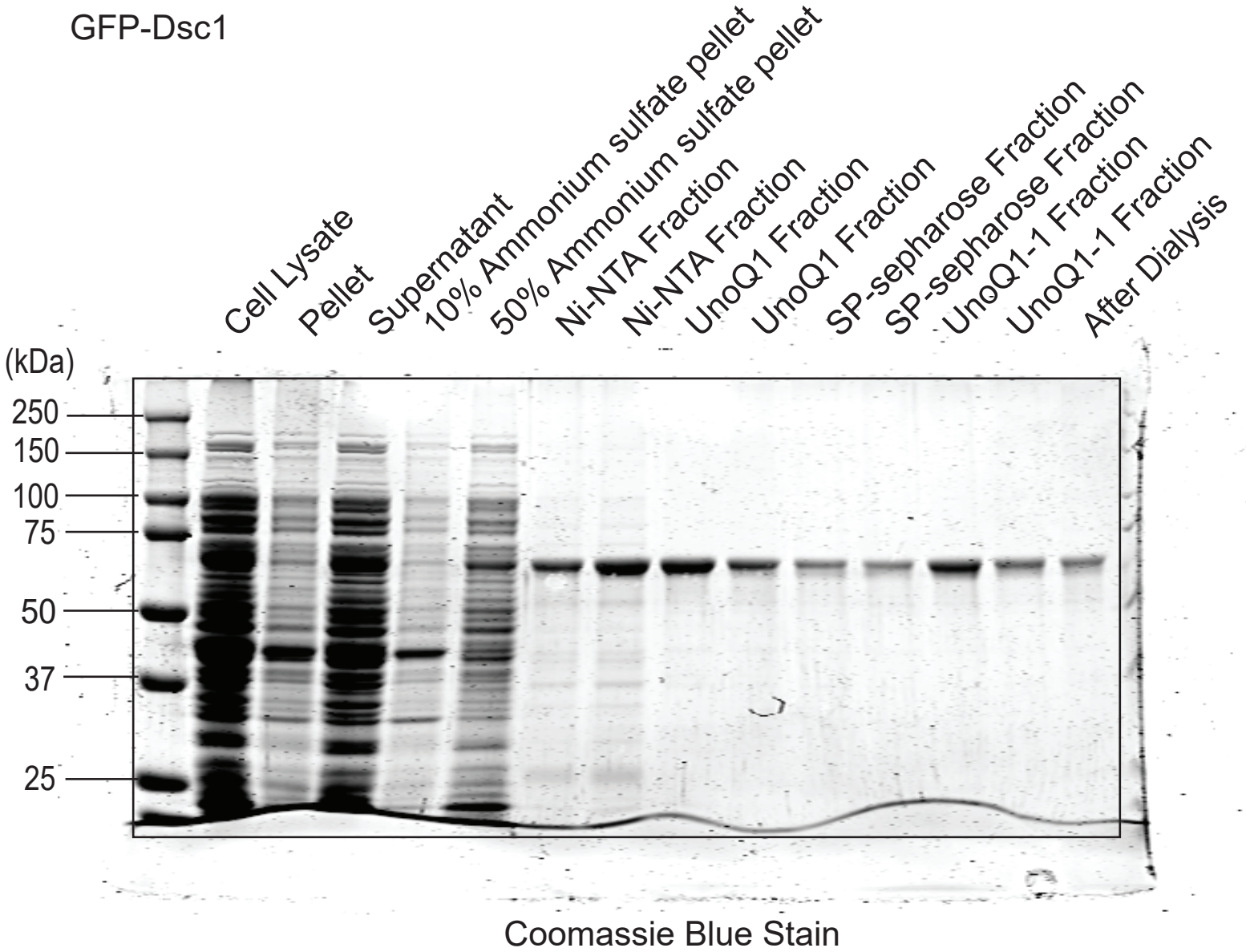
GFP-Dsc1
Cell Lysate
Pellet
Supernatant
10% Ammonium sulfate pellet
50% Ammonium sulfate pellet
Ni-NTA Fraction
Ni-NTA Fraction
UnoQ1 Fraction
UnoQ1 Fraction
SP-sepharose Fraction
SP-sepharose Fraction
UnoQ1-1 Fraction
UnoQ1-1 Fraction
After Dialysis
(kDa)
250
150
100
75
50
37
25
Coomassie Blue Stain

mCh-CortI

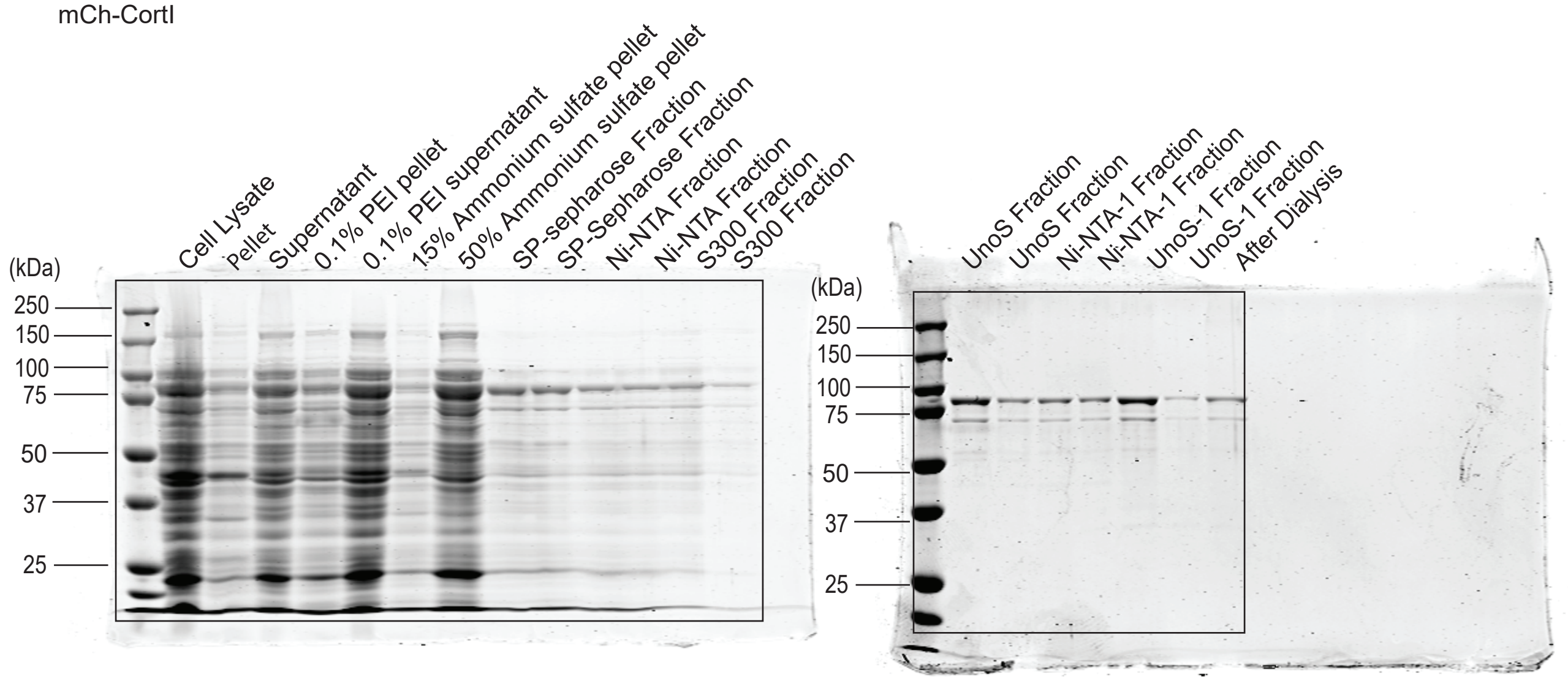

Coomassie Blue Stain
