# Supplementary material for: The effects of pH on cortex and condensate formation
Source: J Cell Biol. 2026 Oct 16;225(12):e202603108. doi: 10.1083/jcb.202603108 (PMC13674344; doi:10.1083/jcb.202603108)
Supplement: Supplementary file 8 [file jcb_202603108_tables1.docx]

| Table S1: List of *Dictyostelium discoideum* stains used throughout this study. | |
| --- | --- |
| Strain | Genotype |
| WT | KAx3 |
| WT: pHluorin-mCherry | KAx3:: pHluorin-mCh, G418^R^ :pDM181 |
| WT: pHluorin-mCherry-CortI | KAx3:: pHluorin-mCh-CortI, G418^R^ :pDM181 |
| WT: cAR1-pHluorin-mCherry | KAx3:: cAR1-pHluorin-mCh, G418^R^ :pDM181 |
| *myoII^-^:* GFP | *myoII^-^*:: GFP, G418^R^ :pDM181 |
| *myoII^-^:* GFP-myoII | *myoII^-^*:: GFP-myoII, G418^R^ :pBIG |
| *cortI^-^:* GFP | *cortI^-^*:: GFP, G418^R^ :pDM181 |
| *cortI^-^:* GFP-CortI | *cortI^-^*:: GFP-CortI, G418^R^ :pExp4 |
| *nhe1^-^* | (Ax2) *nhe1^-^* |
